# Amphiregulin orchestrates the paracrine immune-suppressive function of amniotic-derived cells through its interplay with COX-2/PGE_2_/EP4 axis

**DOI:** 10.1016/j.isci.2024.110508

**Published:** 2024-07-14

**Authors:** Giuseppe Prencipe, Adrián Cerveró-Varona, Monia Perugini, Ludovica Sulcanese, Annamaria Iannetta, Arlette Alina Haidar-Montes, Johannes Stöckl, Angelo Canciello, Paolo Berardinelli, Valentina Russo, Barbara Barboni

**Affiliations:** 1 Unit of Basic and Applied Sciences, Department of Biosciences and Agro-Food and Environmental Technologies, University of Teramo, 64100 Teramo, Italy; 2 Department of Bioscience and Agro-Food and Environmental Technology, University of Teramo, Teramo, Italy; 3 Centre for Pathophysiology, Infectiology and Immunology, Institute of Immunology, Medical University of Vienna, Vienna 1090, Austria

**Keywords:** Cell biology, Immune response, Molecular biology

## Abstract

The paracrine crosstalk between amniotic-derived membranes (AMs)/epithelial cells (AECs) and immune cells is pivotal in tissue healing following inflammation. Despite evidence collected to date, gaps in understanding the underlying molecular mechanisms have hindered clinical applications. The present study represents a significant step forward demonstrating that amphiregulin (AREG) orchestrates the native immunomodulatory functions of amniotic derivatives via the COX-2/PGE_2_/EP4 axis. The results highlight the immunosuppressive efficacy of PGE_2_-dependent AREG release, dampening PBMCs’ activation, and NFAT pathway in Jurkat reporter cells via TGF-β signaling. Moreover, AREG emerges as a key protein mediator by attenuating acute inflammatory response in *Tg(lysC:DsRed2)* zebrafish larvae. Notably, the interplay of diverse COX-2/PGE_2_ pathway activators enables AM/AEC to adapt rapidly to external stimuli (LPS and/or stretching) through a responsive positive feedback loop on the AREG/EGFR axis. These findings offer valuable insights for developing innovative cell-free therapies leveraging the potential of amniotic derivatives in immune-mediated diseases and regenerative medicine.

## Highlights

• Amniotic-derived AEC/AM AREG’s release is dependent on COX-2/PGE_2_/EP4 axis
• AREG orchestrates the paracrine immunomodulatory function of AEC/AM via TGF-β
• AEC/AM adapt to LPS and stretching stimuli by modulating the HIPPO pathway
• Findings provide insights for cell-free therapies using amniotic derivatives in medicine

## Graphical abstract

**Figure:**
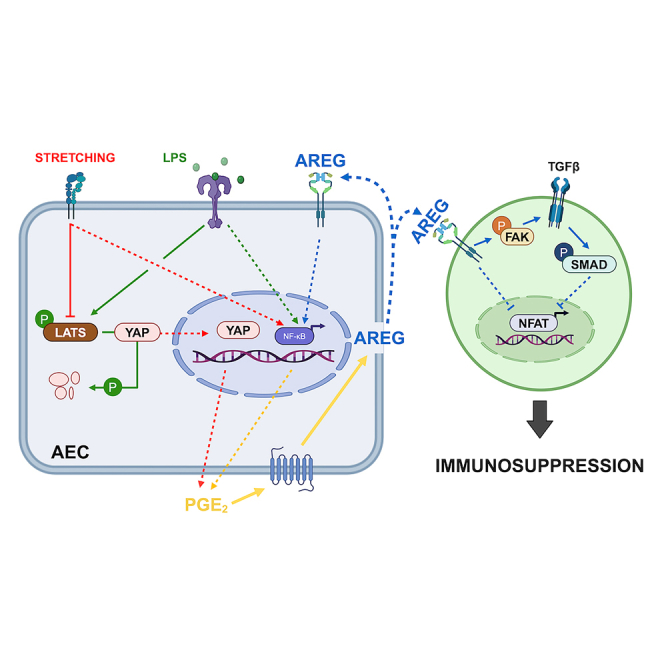

## Introduction

Inflammation is a natural response to injury, playing a crucial role in the initial stages of tissue regeneration. It orchestrates the coordinated recruitment of different types of immune cells to the damaged area, as an adaptive response to restore homeostasis through active communication with stem/stromal and resident cells of local districts.^1^ However, the benefits of a controlled inflammatory phase hinge on a delicate balance of timing and cell involvement. When this equilibrium is disrupted, it can lead to the development of degenerative, inflammatory-based diseases, making chronic inflammation a potentially detrimental force that perpetuates tissue remodeling and functional impairment, ultimately resulting in fibrosis^2^ and autoimmune diseases.^3^

In this complex scenario, stem cells, particularly amniotic-derived cells like amniotic epithelial cells (AECs), have emerged as a promising avenue due to their remarkable plasticity and immunomodulatory properties.^4^^,^^5^ AECs, derived from the amniotic membrane (AM), the innermost layer of the placenta, exhibit a unique combination of embryonic and adult stem cell features, positioning them as a versatile and reliable source for regenerative therapies.^6^ Their exceptional differentiative plasticity enables them to differentiate into specific cell lineages, offering compelling prospects for tissue repair and regeneration.^7^^,^^8^^,^^9^^,^^10^ Moreover, AECs and AM have shown an immune-modulating function through the precise release of immunomodulatory molecules, effectively reshaping the inflammatory environment, and promoting tissue regeneration.^11^^,^^12^^,^^13^^,^^14^

Taking advantage of this feature, AEC-conditioned media (CM) has emerged as a promising cell-free approach, leveraging the array of growth factors, cytokines, and extracellular vesicles released by these cells.^15^ AEC’s CM plays a crucial role in modulating immune cell activities by suppressing the proliferation of peripheral blood mononuclear cells (PBMCs),^16^^,^^17^^,^^18^^,^^19^ influencing the recruitment, maturation, and proliferation of antigen-presenting cells, such as macrophages and dendritic cells,^19^^,^^20^^,^^21^^,^^22^ and inhibiting T cell activation and proliferation.^18^ Ongoing investigations are unraveling the precise mechanisms underlying the pleiotropic immune paracrine role of amniotic cells/tissues and derivatives, with active validation through clinical trials for potential applications in immune-based disorders.^3^ This progress represents a significant step toward translating the promising therapeutic potential of AEC CM into clinical reality, offering innovative avenues for addressing immune-related conditions through cell-free approaches.

In this context, Rossi et al. demonstrated the intrinsic anti-proliferative impact of CM, derived from term human amniotic cells or the whole membrane, on PBMCs.^18^ This influence was found to be further potentiated when cells were exposed to external stimuli mimicking inflammatory conditions.^18^ The anti-proliferative efficacy of amniotic-derived cells and tissues is predominantly mediated through soluble factors, manifesting their effects in both cell to cell contact and *trans*-well co-cultures.^9^^,^^23^^,^^24^^,^^25^ Moreover, the same authors initiated to unravel the molecular mechanisms underpinning this immunomodulatory function. Prostaglandin E2 (PGE_2_) has been identified as a key molecule responsible for a substantial portion of the anti-proliferative effects observed in term amniotic derivatives, instead of interleukin-6 (IL-6) and interleukin-10 (IL-10).^18^ Interestingly, indoleamine 2,3-dioxygenase (IDO), nitric oxide (NO), transforming growth factor β (TGF-β), and hepatocyte growth factor (HGF) commonly implicated in mesenchymal stem cells (MSCs) from other sources, were not identified as significant contributors.^18^ Moreover, PGE_2_ was found to engage in a vast interconnected molecular network controlling the modulation of several other crucial immune-modulatory proteins, including matrix-metalloproteinase 9 (MMP-9),^26^ interleukin-4 (IL-4),^27^ and amphiregulin (AREG).^28^

An increasing interest is turning toward the interaction between PGE_2_ and AREG, an established crosstalk between stem and immune cells in tailoring the immune microenvironment after injuries.^28^^,^^29^ While AREG and other epithelial growth factor (EGF) family members are originally described as epithelial cell-derived factors highly active in female reproductive tissues,^30^^,^^31^^,^^32^^,^^33^ recent data show that this protein can be expressed by multiple populations of activated immune cells in a variety of inflammatory conditions, mediating the crosstalk between immune and epithelial cells during tissue homeostasis recovery.^34^^,^^35^ Recent studies, indeed, indicate AREG as a primary factor in promoting the restoration of tissue integrity following damage associated with both acute or chronic inflammation.^34^^,^^36^^,^^37^

Starting from these premises, the present study aimed to investigate whether an interaction exists between cyclooxygenase 2 (COX-2)/PGE_2_/prostaglandin receptor 4 (EP4) and AREG/EGF receptor (EGFR) axes and what role it plays in the paracrine immunosuppressive activities of amniotic derived cell/tissue, by deepening the involved intracellular signaling pathway.

Here, AREG is demonstrated for the first time to be constitutively secreted from both AEC and AM, exerting a prominent immunosuppressive role *in vitro* via TGF-β on PBMCs and Jurkat cells, influencing their activity and modulating the intracellular nuclear factor of activated T cell (NFAT) pathway, respectively, and *in vivo* on tail fin transected zebrafish larvae, orchestrating the recruitment and migration of immune cells toward the wounded area. Remarkably, AREG secretion in response to AM exposure to specific signals, lipopolysaccharides (LPS), and stretching (mimicking pro-inflammatory and middle stage of gestation environments, respectively) is entirely dependent on PGE_2_ release. The results indicate that PGE_2_ operates as a co-responsible factor rather than a critical immunomodulatory agent in the amniotic-derived cell/AM system. Interestingly, CM obtained from both AEC and AM does not reach concentrations of PGE2 high enough to significantly inhibit immune cell function. However, these CM prove highly effective in modulating the release of AREG. Furthermore, the investigation revealed two distinct downstream alternative responses of AM exposed to LPS or stretching inputs. These mechanisms act by activating or blunting the Hippo pathway, respectively, tuning the COX-2/PGE_2_/EP4 signaling axis via the yes-associated protein (YAP).

## Results

### Amniotic-derived cells/tissue’ CM effectively suppressed PBMCs activation and Jurkat NFAT signaling pathway

The study firstly assessed the immunomodulatory influence of CM derived from AEC and AM (under native vs. stimulatory conditions) using the MTS PBMC activation test.

**Figure 1:**
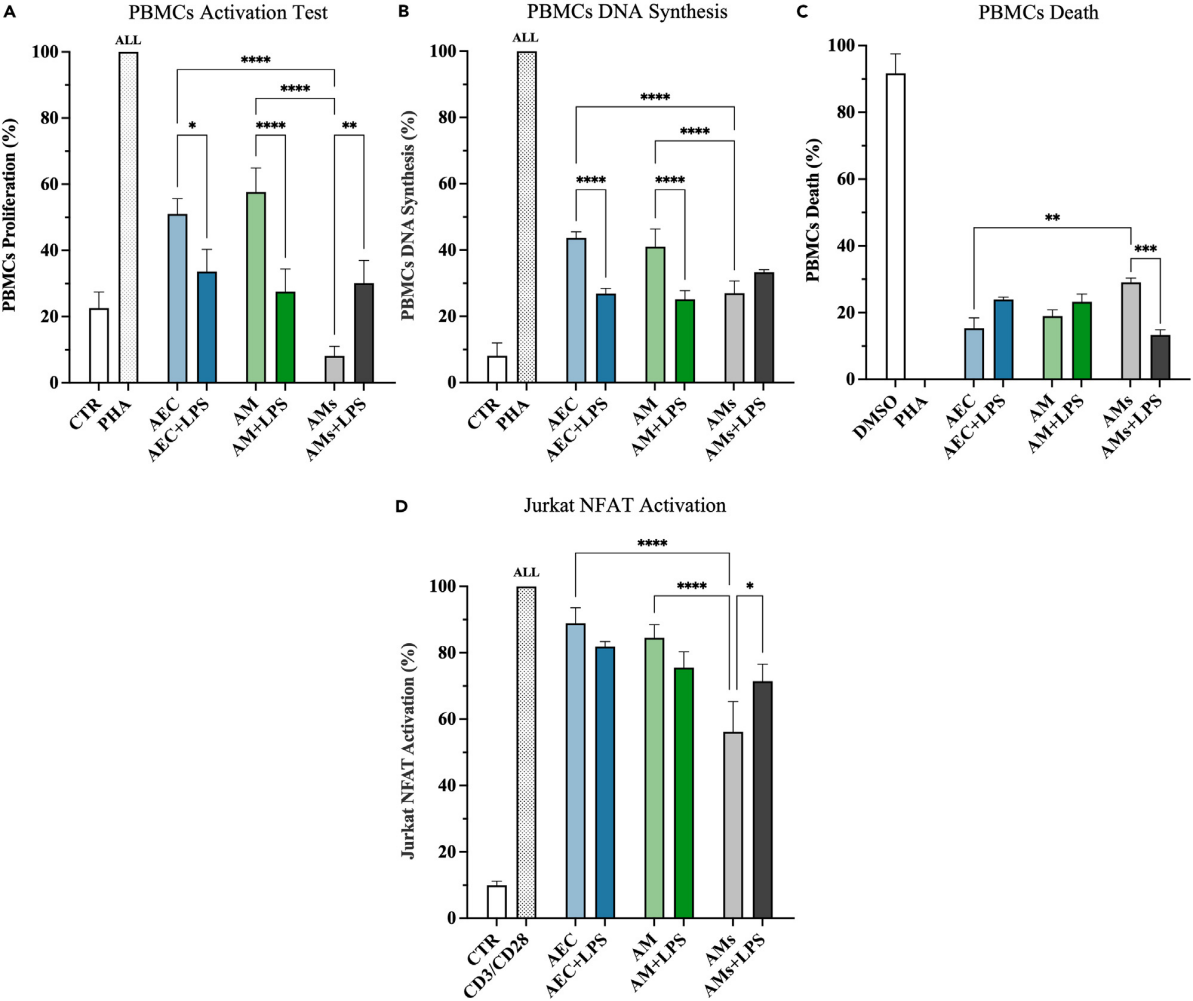
Amniotic-derived cells/tissue’ CM effectively suppressed PBMCs activation and Jurkat NFAT signaling pathway (A–C) PHA-stimulated PBMCs were treated with the different CM (±LPS) for 48 h and assessed for their: (A) proliferation, (B) DNA synthesis, and (C) cell death. Data were normalized on PHA-stimulated PBMCs (100% of proliferation). (D) CD3/CD28-stimulated Jurkat reporter cells were evaluated for the inhibition of NFAT activation after treatment with the different CM (±LPS) for 48 h. Data are normalized on CD3/CD28 stimulated Jurkat (100% of NFAT activation). Data (mean ± SD) represent 3 independent sets of experiments (*n* = at least 3 biological replicates in each group per set; each biological replicate assayed in at least 3 technical replicates). All, ∗, ∗∗, ∗∗∗, and ∗∗∗∗ Statistically significant values between the different studied groups (*p* < 0.05, *p* < 0.01, *p* < 0.001, and *p* < 0.0001, respectively).

As illustrated in Figure 1A, whole amniotic-derived CM inhibited the proliferation of PBMCs induced through phytohemagglutinin (PHA) (*p* < 0.01 vs. PHA).^16^^,^^17^ However, the immunosuppressive action was strictly related to CM’ source. More in detail, the most effective CM was derived from stretched AM (AMs) bringing inhibition over 90% (*p* < 0.0001 vs. both AEC and AM; Figure 1A). Instead, the immune paracrine activity of both AEC and AM was significantly enhanced by LPS’s pro-inflammatory mimicking stimulus (*p* < 0.05 and *p* < 0.0001 vs. AEC+LPS and AM+LPS, respectively; Figure 1A).^16^^,^^17^ Of note, LPS and stretching did not synergize in boosting the paracrine response of AM that, on the contrary, was significantly reduced (AMs vs. AMs+LPS, *p* < 0.01; Figure 1A). This reduction was not due to a deleterious effect of the combined stimuli on the cell viability on the membranes (data not shown).

To verify the cellular mechanisms involved in PBMCs inhibition, the effects of CM exposure were analyzed at either DNA synthesis or apoptosis level. As summarized in Figures 1B and 1C, both DNA synthesis and apoptosis profiles were coherent with the inhibitory influences of CM on PBMCs activation (Figure 1A). However, CM is mainly operated by inhibiting DNA synthesis rather than inducing apoptosis. More in detail, cell cycle block was always induced in PBMCs exposed to CM (AMs vs. AEC and AM, *p* < 0.0001; Figure 1B). Of note, it was more efficient using AEC+LPS and AM+LPS-derived CM (*p* < 0.0001 vs. AEC and AM, respectively; Figure 1B). On the contrary, the induction of apoptosis became prominent in the stretching condition (*p* < 0.001 AMs vs. AMs+LPS; Figure 1C).

Therefore, the immunosuppressive influence of CM on PHA-activated PBMCs is regulated by the convergence of combined cellular mechanisms: predominantly through the inhibition of proliferation but also through the induction of apoptosis.

Then, the study moved to verify whether the paracrine molecules released from AEC and AM were also able to interfere with specific intracellular immune pathways.^38^^,^^39^^,^^40^^,^^41^ To this aim, a xeno *in vitro* setting has been designed using human Jurkat reporter cells to test the NFAT pathway after CD3/CD28 activation. Jurkat-derived reporter systems are frequently employed to investigate how external stimuli influence the activation of transcription factors like NFAT.^42^ The regulation of NFAT proteins is governed by intracellular Ca^2+^ signaling, playing a crucial role in T cell activation, differentiation, and self-tolerance.^43^^,^^44^ The results demonstrated that all CM were able to inhibit NFAT activation induced by CD3/CD28 exposition (*p* < 0.01 vs. CTR; Figure 1D). Moreover, AMs’ CM still expressed a higher inhibitory influence (*p* < 0.0001, *p* < 0.0001, and *p* < 0.05 vs*.* AEC, AM, and AMs+LPS, respectively; Figure 1D).

In conclusion, the amniotic-derived CM constitutively exert an immunosuppressive influence that can be strongly enhanced when the cell/tissues are exposed to external stimuli, in particular, stretching. The underlying immunosuppressive mechanisms of CM recognize either the modulation of specific intracellular signaling pathways (NFAT) or the fine-tuning balance between immune cell proliferation and apoptosis.^38^^,^^39^^,^^40^^,^^41^^,^^45^^,^^46^^,^^47^

### AREG emerged as the key players in CM’ immunosuppressive role on PBMCs

The second phase of the study was addressed to demonstrate the role exerted by PGE_2_ and AREG, two major paracrine crosstalking immune factors in other stem^18^^,^^29^^,^^35^^,^^48^ and cancer cells.^49^^,^^50^^,^^51^^,^^52^^,^^53^^,^^54^^,^^55^^,^^56^

**Figure 2:**
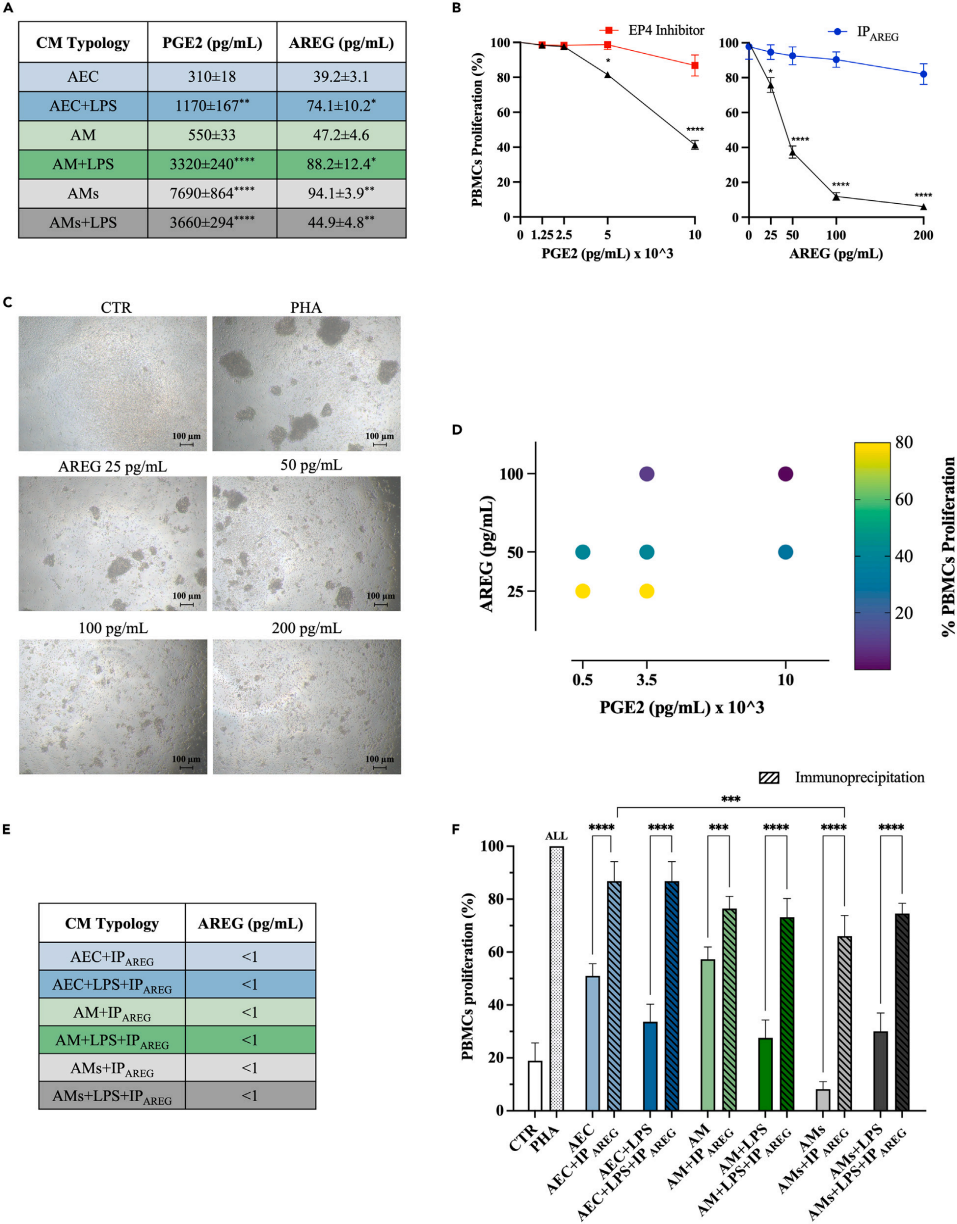
AREG emerged as the key players in CM’ immunosuppressive role on PBMCs (A) ELISA for secreted levels of PGE_2_ and AREG in the different CM (±LPS). (B) PGE_2_ and AREG dose-response curves for PHA stimulated PBMCs proliferation (48 h). (C) Representative phase-contrast images of PHA stimulated PBMCs AREG dose-response test. (D) PCA plot of PHA stimulated PBMCs proliferation treated with different concentrations of PGE2 and AREG like those recorded under physiological conditions. The first two principal components (PGE_2_ and AREG concentrations) are plotted and colored according to PBMCs proliferation rate (as ratio (%) to PHA stimulated PBMCs). PCA was performed using all analyzed data. (E and F) AREG ELISA in the CM after AREG IP, (F) subsequently tested on PHA-stimulated PBMCs (activation test after 48 h of culture). Data are shown as a ratio (%) to PHA-stimulated PBMCs. Data (mean ± SD) represent 3 independent sets of experiments (*n* = at least 3 biological replicates in each group per set; each biological replicate assayed in at least 3 technical replicates). All, ∗, ∗∗, ∗∗∗, and ∗∗∗∗ Statistically significant values between the different studied groups (*p* < 0.05, *p* < 0.05, *p* < 0.01, and *p* < 0.0001, respectively). Scale bar, 100 μm.

The levels of both the molecules on the CM changed according to AEC and AM cultural conditions with higher concentration in CM having the greatest immunosuppressive influence (AEC vs. AEC+LPS and AM vs. AM+LPS, AMs and AMs+LPS, *p* < 0.05; Figure 2A). More in detail, PGE_2_ content showed a dramatic increase in CM collected from AEC and AM after exposure to LPS (4- and 6-fold increase, respectively, *p* < 0.01; Figure 2A) or stretching (AMs more than 10 and 6-fold increase vs*.* AEC and AM, respectively, *p* < 0.0001; Figure 2A). The combination of LPS and stretching stimuli again induced a detrimental influence (AMs vs. AMs+LPS, *p* < 0.0001; Figure 2A), even if AMs+LPS-derived CM still conserved very high levels of PGE_2_ (vs*.* AEC+LPS, *p* < 0.0001; Figure 2A) similar to those recorded in CM obtained from AM+LPS (*p* > 0.05; Figure 2A).

Moreover, AREG levels followed a similar trend: LPS or stretching inputs were able to significantly boost the release of AREG by doubling the protein content of their CM (*p* < 0.05; Figure 2A). Once again, LPS stimulation did not exert any further stimulation on AREG secretion in AMs (AMs vs. AMs+LPS, *p* < 0.0001; Figure 2A).

To study the immunosuppressive role of AREG and PGE_2_, two dose-response tests were performed (Figure 2B) by supplementing PBMCs with concentrations mimicking those recorded in CM: from 0 to 10.000 pg/mL for PGE_2_ and from 0 to 200 pg/mL for AREG. Of note, PGE_2_ supplementation started to exhibit a significant inhibitory effect when the medium concentration exceeded 5,000 pg/mL (*p* < 0.05 vs. 0 pg/mL; Figure 2B), a level recorded exclusively in the AMs-derived CM (< 8.000 pg/mL; Figure 2A). On the contrary, PBMCs were highly sensitive to the immunosuppressive influence of AREG (Figure 2B). More in detail, 25 pg/mL of AREG induced 20% of PBMC immunosuppression. Of note, this concentration was below AEC and AM protein basal release (Figure 2A). Moreover, more than 85% of PBMCs activation was prevented using 100 pg/mL of AREG (*p* < 0.001 vs. 0 pg/mL; Figures 2B and 2C), thus reproducing a dose comparable to AM+LPS and AMs CM content (Figure 2A).

The inhibitory influences of PGE_2_ and AREG on PBMCs activation were, further, confirmed by designing a dose-response curve in the presence of the prostaglandin E2 receptor 4 (EP4) inhibitor (EP4_IN_)^29^ or by immunoprecipitating AREG content (IP_AREG_), respectively. In both the experimental setups, PGE_2_ and AREG inhibitory action on PBMCs were drastically prevented (*p* > 0.05 vs. PHA-activated PBMCs; Figures 2B and 2C).

Then, PGE_2_ and AREG composition of CM was pharmacologically reproduced by supplementing them in culture media at the previously recorded concentrations. The principal-component analysis (PCA) revealed that the immunosuppressive effect of CM was mainly mediated from AREG, prevailing at lower doses (Figure 2D).

Finally, to further validate the pivotal immunomodulatory role of AREG, the CM protein content was depleted through immunoprecipitation (IP_AREG_). CM lacking AREG (Figure 2E) drastically lost their inhibitory influence (*p* < 0.001; Figure 2F). The IP_AREG_ effect was maximized in CM derived from AEC and AEC+LPS where only 15% of the inhibitory influence on PBMC persisted, against 20% of AM±LPS or 30% of AMs+LPS and AMs, respectively (*p* < 0.001 AEC+IP_AREG_ vs. AMs+IP_AREG_; Figure 2F).

In conclusion, the inhibitory action of AEC/AM-derived CM is mainly ascribable to AREG, although PGE_2_ was documented to exert an immunomodulatory action in other cell models.^18^^,^^34^^,^^35^

### AREG is a key immune modulator of acute inflammation induced in zebrafish larvae

Afterward, an *in vivo* study was designed to elucidate whether AREG conserved the immunosuppressive role during a process of acute inflammation exploiting zebrafish larvae as a robust model. To this aim, tail fin amputation was performed to induce an acute inflammatory response in transgenic zebrafish *Tg(lysC:DsRed2)* larvae expressing a red fluorescent protein, thus documenting *in vivo* the effect of the CM on the mobilization and proliferation of immune cells expressing lysozyme.

**Figure 3:**
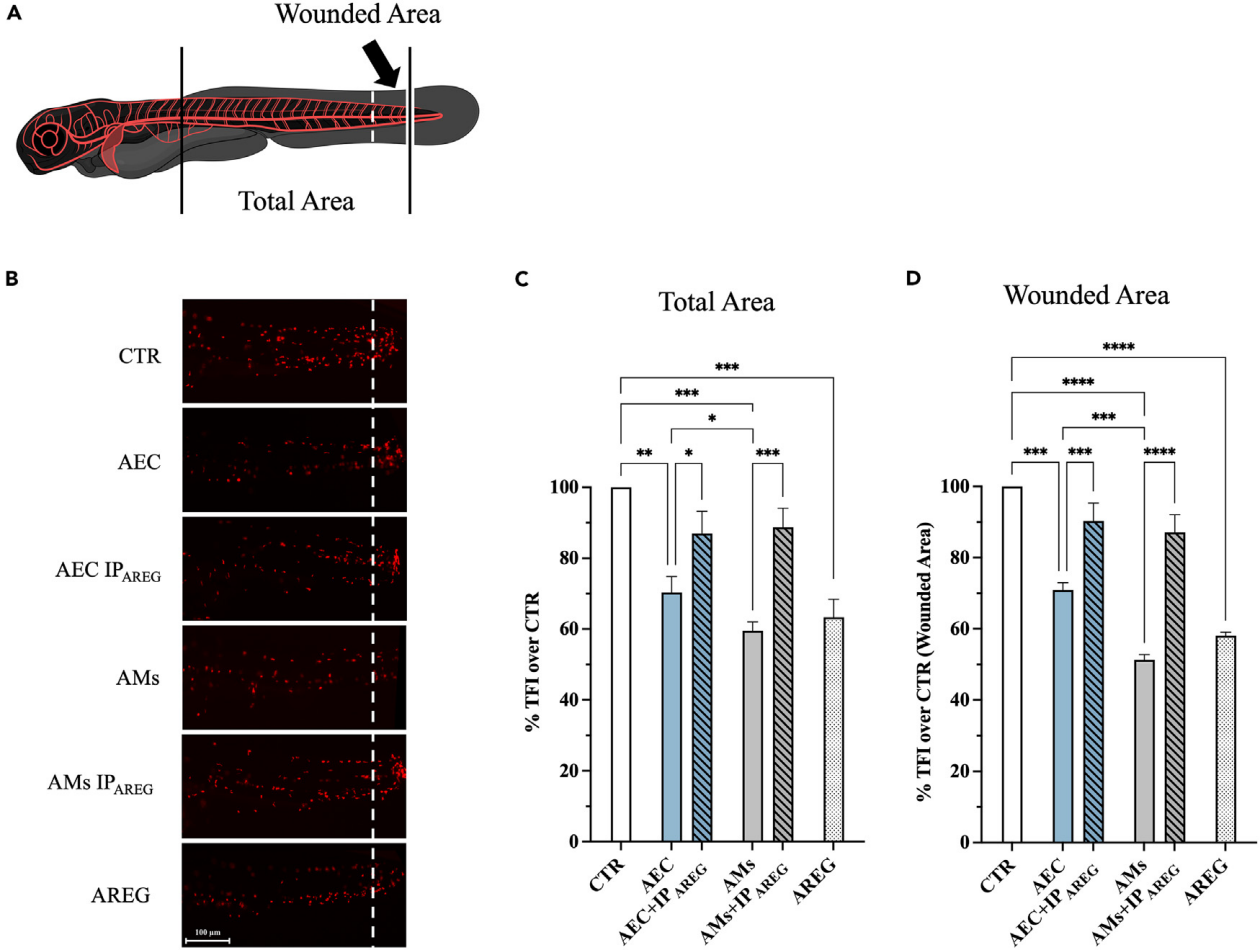
AREG is a key immune modulator of acute inflammation induced in zebrafish larvae (A) Illustration depicting a zebrafish larva at 72 h post-fertilization (hpf). (B) Exemplary images of Tg(lysC:DsRed2) larvae post tail fin dissection, showcasing the localization of immune cells in the tail and their accumulation at the site of injury 48 h post treatment (120 hpf) (right of the dashed white line). (C and D) Densitometric analysis of total fluorescence intensity (TFI), presenting the percentage of immune cell proliferation detected in the tail and (D) within the wounded area. Data (mean ± SD) represent 3 independent sets of experiments (*n* = at least 3 biological replicates in each group per set). ∗, ∗∗, ∗∗∗, and ∗∗∗∗ Statistically significant values between the different studied groups (*p* < 0.05, *p* < 0.01, *p* < 0.001, and *p* < 0.0001, respectively). Scale bar, 100 μm.

In detail, transgenic zebrafish larvae, at 72 h post-fertilization, were exposed to CM derived from AEC or AMs (CM with the lowest and highest AREG content, respectively) after caudal fins amputation for 48 h (120 hpf) (Figure 3A). To study the AREG role, CM were also deprived of protein by immunoprecipitation (AEC+IP_AREG_ and AMs+IP_AREG_, respectively) or exogenous AREG was supplemented at high concentration (90 pg/mL) to standard cultural medium. Total fluorescence intensity (TFI) was quantified on the whole animal body or on the wounded area, respectively, to assess the immune cell response.

Of note, CM derived from AEC and AMs displayed both an inhibitory influence on immune cell recruitment by significantly reducing TFI either in wounded area or in overall animal (*p* < 0.05 vs. CTR; Figures 3C and 3D). Furthermore, the strongest immunosuppressive influence of CM derived from AMs was also confirmed under the *in vivo* zebrafish model of acute inflammation (*p* < 0.05 vs. AEC; Figures 3C and 3D). The key role of AREG in mediating this paracrine action of both AEC and AMs was strongly confirmed by the experiments of IP_AREG_ which resulted in the immuno-suppression reduction ranging from 20 to 30%. The *in vivo* immunosuppressive role of AREG was, finally, demonstrated by the pharmacological addition the protein (Figures 3B and 3D). Of note, AREG supplementation determined a significant reduction of TFI with fold change similar to that of AMs-derived CM (*p* > 0.05; Figures 3C and 3D). Overall, these results highlight how the immunosuppressive action of CM is operative also *in vivo* and strictly AREG dose-dependent (AEC+IP_AREG_/AMs+IP_AREG_ < AEC ≤ AMs/90 pg/mL AREG).

In conclusion, both AEC- and AMs-derived CM exerts an AREG-mediated- immunosuppressive effect expressed in zebrafish larvae by inhibiting either immune cell proliferation (overall effect) or cell recruitment in the amputated tail (wound area influence).

### Amniotic-derived cells/tissue AREG release is dependent on COX-2/PGE_2_/EP4 axis

Starting from the evidence that PGE_2_ and AREG levels in CM follow a similar pattern, the synergic mechanisms of release were investigated.

**Figure 4:**
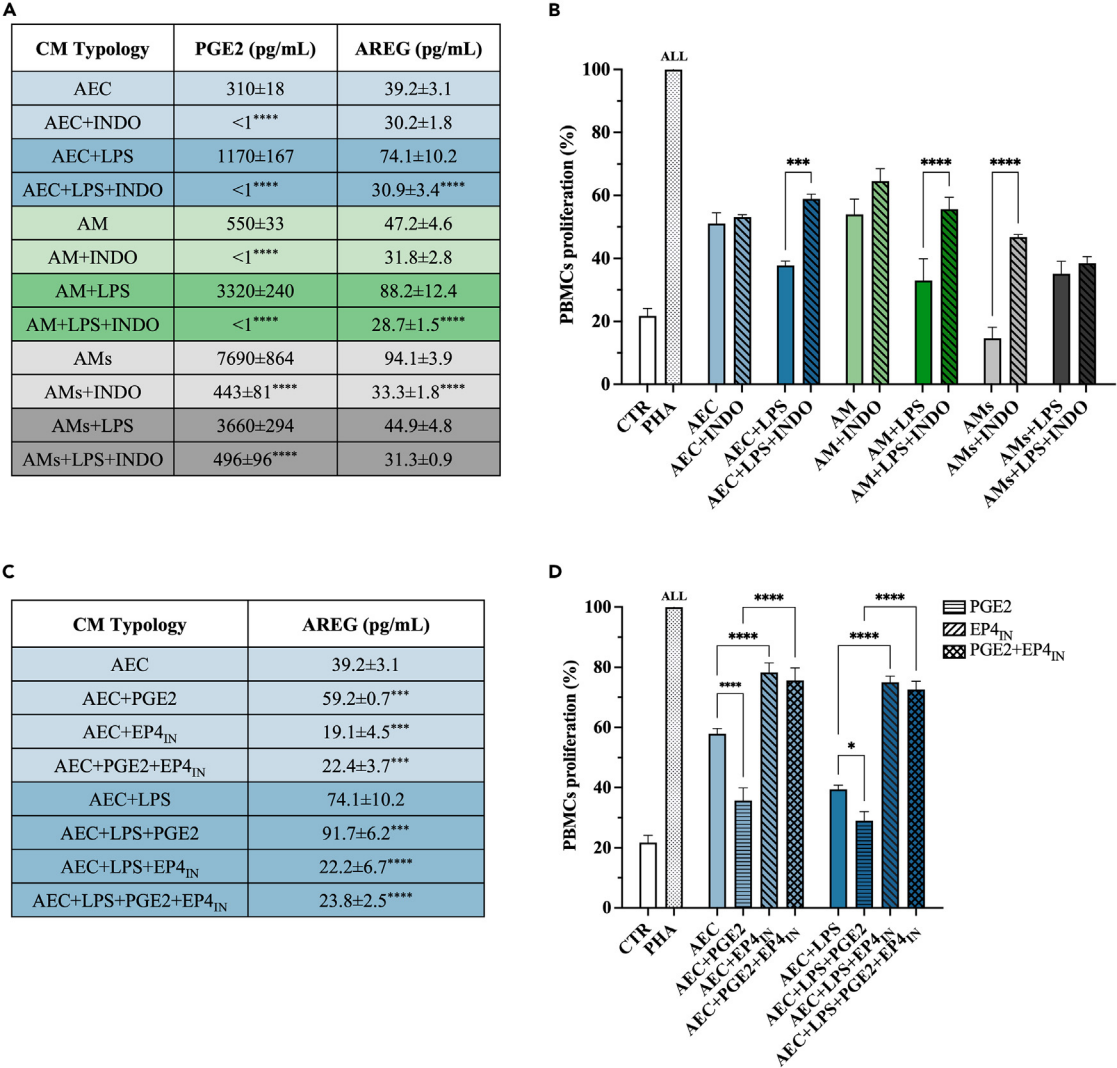
Amniotic-derived cells/tissue AREG release is dependent on COX-2/PGE_2_/EP4 axis (A) PGE_2_ and AREG ELISA in the CM (±LPS) obtained from amniotic-derived cells/tissue cultured with INDO for 24h. (B) CM (±LPS) obtained from amniotic-derived cells/tissue cultured with INDO for 24 h were tested on PHA-stimulated PBMCs (activation test after 48 h of culture). Data are shown as a ratio (%) to PHA-stimulated PBMCs. (C) AREG ELISA in the CM (±LPS) obtained from amniotic-derived cells/tissue cultured with PGE_2_ or with EP4 inhibitor for 24h. (D) CM (±LPS) obtained from amniotic-derived cells/tissue cultured with PGE_2_ or EP4 inhibitor for 24 h were tested on PHA-stimulated PBMCs (activation test after 48 h of culture). Data are shown as a ratio (%) to PHA-stimulated PBMCs. Data (mean ± SD) represent 3 independent sets of experiments (*n* = at least 3 biological replicates in each group per set; each biological replicate assayed in at least 3 technical replicates). All, ∗, ∗∗∗, and ∗∗∗∗ Statistically significant values between the different studied groups (*p* < 0.01, *p* < 0.05, *p* < 0.001, and *p* < 0.0001, respectively).

The first experimental step was to block the synthesis of PGE_2_ by culturing amniotic-derived cells/tissue with indomethacin (INDO). The use of this cyclooxygenase inhibitor was highly effective on PGE_2_ release. Indeed, INDO totally abolished PGE_2_ secretion in AEC and AM (<1 pg/mL; Figure 4A), while led to a significant reduction (7 and 17-fold) in AMs, settling CM content on 443 ± 81 pg/mL and 496 ± 96 pg PGE_2_/mL w/wo LPS, respectively (*p* < 0.0001 vs. without INDO; Figure 4A).

Interestingly, the use of the cyclooxygenase inhibitor had a relevant indirect impact on the release of AREG. More precisely, INDO exposure significantly prevented LPS or stretching boost on AREG release (in AEC and AM∼30 pg/mL). More in detail, the treatment determined a 2- and a 3-fold AREG secretion reduction in AEC+LPS and AM+LPS/AMs, respectively (for all *p* < 0.001 Figure 4A).

Of note, INDO treatment did not exert any influence on the basal secretion of AEC and AM (*p* > 0,05, respectively) as well as the inhibitor was not effective for AMs+LPS AREG release (Figure 4A).

The PBMC activation test, performed using the CM collected from AEC or AM treated with INDO, indirectly confirmed the critical immunomodulatory role of AREG. Indeed, the immunosuppressive influence of CM resulted unaffected by INDO treatment (*p* > 0.05 vs. without INDO; Figure 4B) exclusively in AEC, AM, and AMs+LPS, all CM where the inhibitor significantly reduced PGE_2_ without affecting AREG *(*Figure 4A). On the contrary, a significant loss of immunosuppressive CM activity was recorded in AEC+LPS, AM+LPS, and AMs (Figure 4B), CM where INDO treatment induced a synergic drop in both PGE_2_ and AREG levels (Figures 4A and 4B).

To confirm the influence of PGE_2_ in enhancing AREG release, a further experimental setup was designed by supplementing additional PGE_2_ during AEC/AM culture. To confirm the influence of PGE2 in enhancing AREG release, a further experimental setup was designed by supplementing additional PGE2 during AEC/AM culture. Given the limitations of using a silencing approach via siRNA on the ovine model, the investigation of the interaction between AREG and PGE2 was ensured through two rigorous systems: INDO, as previously discussed, and PGE2 supplementation and EP4 inhibitor. Therefore, to reproduce the high PGE_2_ concentration recorded in AM+LPS or AMs-derived CM, 5000 pg/mL of PGE_2_ were supplemented to AEC (AEC+PGE_2_ and AEC+LPS+PGE_2_; Figure 4A). Of note, the AEC cultures in the media supplemented with PGE_2_ enhanced AREG release. In detail, the AREG levels in CM derived from AEC+PGE_2_ and AEC+LPS+PGE_2_ were 1.5 and 1.2 times higher, respectively (*p* < 0.001 vs. without exogenous PGE_2_; Figure 4C). Of note, PGE_2_ positive feedback on AREG secretion was mainly mediated by the EP4 receptor. Indeed, the presence of EP4_IN_ prevented the positive influence of PGE_2_ supplementation by significantly decreasing AREG levels (*p* < 0.001 vs. AEC+PGE_2_ and *p* < 0.0001 vs. AEC+LPS+PGE_2_; Figure 4C) below the basal ones (*p* < 0.001 vs. AEC and *p* < 0.0001 vs. AEC+LPS; Figure 4C).

The inhibition induced by PBMCs activation by CM was strictly dependent on AREG-induced levels (Figure 4D). Notably, the CM derived from AEC supplementation of PGE_2_ significantly increased their inhibition on PBMCs (*p* < 0.0001 vs. AEC and *p* < 0.05 vs. AEC+LPS; Figure 4D). In addition, EP4 inhibitor treatment reverted this PGE_2_ inductive effect on PBMCs (*p* < 0.0001 vs. wo EP4_IN_; Figure 4D).

Altogether the results confirm the pivotal role of AREG in mediating the CM’s immunosuppressive influence on PBMCs, highlighting a co-responsive positive control of COX-2/PGE_2_/EP4 axis on protein release. In particular, PGE_2_/EP4 exerts positive feedback enhancing AREG secretion when AEC/AM are exposed to external stimuli. On the contrary, the basal secretion of AREG from both AEC and AM is independent of PGE_2_ control.

### The immunosuppressive role of AREG is mediated via TGF-β signaling

In order to verify if AREG and TGF-β could interact in mediating the immunomodulatory action of AEC-derived CMs as previously demonstrated in other cell models,^35^^,^^57^ a specific setup of experiments was carried out by assessing the immunomodulatory role of CMs characterized from different content of AREG (absence, extra (100 pg/mL) or basal concentrations) in the presence or absence of 10 ng/mL of TGF-β or a TGF-β inhibitor.

**Figure 5:**
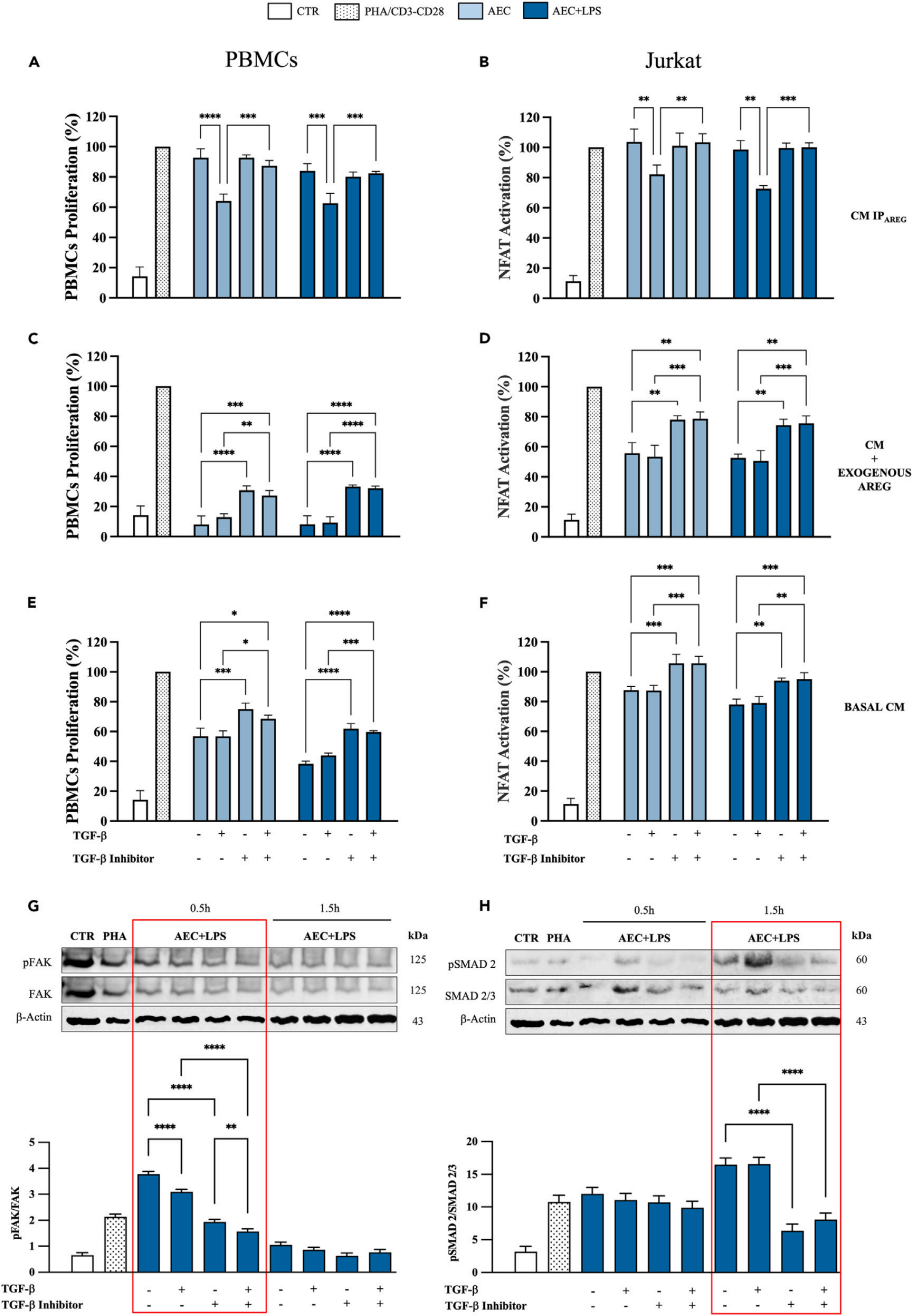
The immunosuppressive role of AREG is mediated via TGF-β signaling (A, C, and E) Proliferation test of PHA-stimulated PBMCs±TGF-β inhibitor/TGF-β were treated with the different CM (±LPS) (A) in the absence of AREG or (C) in its presence in extra (exogenous; 100 pg/mL) or (E) basal concentrations for 48 h. Data were normalized on PHA-stimulated PBMCs (100% of proliferation). (B, D, and F) CD3/CD28-stimulated Jurkat reporter cells were evaluated for the inhibition of NFAT activation after treatment for 48 h with the aforementioned CM. Data are normalized on CD3/CD28 stimulated Jurkat (100% of NFAT activation). (G and H) Representative WB images and relative densitometric analysis of p-FAK (normalized on FAK total content), and of p-SMAD2 (normalized on SMAD2/3 total content) relative to PBMCs cultured with AEC+LPS-derived CM in the presence of TGF-β inhibitor/TGF-β for 0.5 and 1.5 h. Data were normalized on β-actin protein expression. Data (mean ± SD) represent 3 independent sets of experiments (*n* = at least 3 biological replicates in each group per set; each biological replicate assayed in at least 3 technical replicates). All, ∗, ∗∗, ∗∗∗, and ∗∗∗∗ Statistically significant values between the different studied groups (*p* < 0.05, *p* < 0.01, *p* < 0.001, and *p* < 0.0001, respectively).

Overall, the present results confirmed that AREG modulated PBMCs and Jurkat activation acting through TGF-β signaling pathway. In detail, CMs deprived of AREG, lost their immunosuppressive effect, which was restored by the supplementation of exogenous TGF-β (*p* < 0.01 vs. AEC±LPS; Figures 5A and 5B). On the contrary, CMs with extra AREG showed a huge boost in their immunomodulatory activity. The use of TGF-β inhibitor negatively impacted this effect (*p* < 0.01 vs. AEC±LPS; Figures 5C and 5D). Finally, at basal AREG concentrations, the use of TGF-β inhibitor significantly reduced the immune modulatory influence of CMs (*p* < 0.001 vs. AEC±LPS; Figures 5E and 5F), while TGF-β supplementation did not affect it.

Further experiments were carried out to assess the activation of FAK and SMAD2 as two intracellular mediators of TGF-β signaling. Of note, PBMC modulation promoted by AEC+LPS-derived CM is accompanied by FAK activation in 0.5 h followed by SMAD2 phosphorilation (after 1.5 h). Furthermore, the involvement of TGF-β in mediating FAK and SMAD2 activation induced by CMs was confirmed by exposing the immune cells to its inhibitor (Figures 5G and 5H).

These results underscore the role of TGF-β signaling in mediating the immunosuppressive effects of the produced CM. Therefore, the interplay between PGE_2_-driven AREG production and AREG-mediated TGF-β activation via integrins highlights a complex regulatory network essential for immune suppression, where disruption can lead to uncontrolled immune responses and autoimmunity.

### YAP nuclear translocation promoted by stretching mediated the enhanced release of PGE_2_ and AREG

Finally, the research moved toward the comprehension of AM intracellular mechanisms controlling PGE_2_/AREG release in response to simultaneous stretching and LPS external stimulation. Specifically, the role of YAP, a key mechano-transducer intracellular effector, was investigated by considering also the regulatory role documented in other stem cell models.^58^^,^^59^^,^^60^

**Figure 6:**
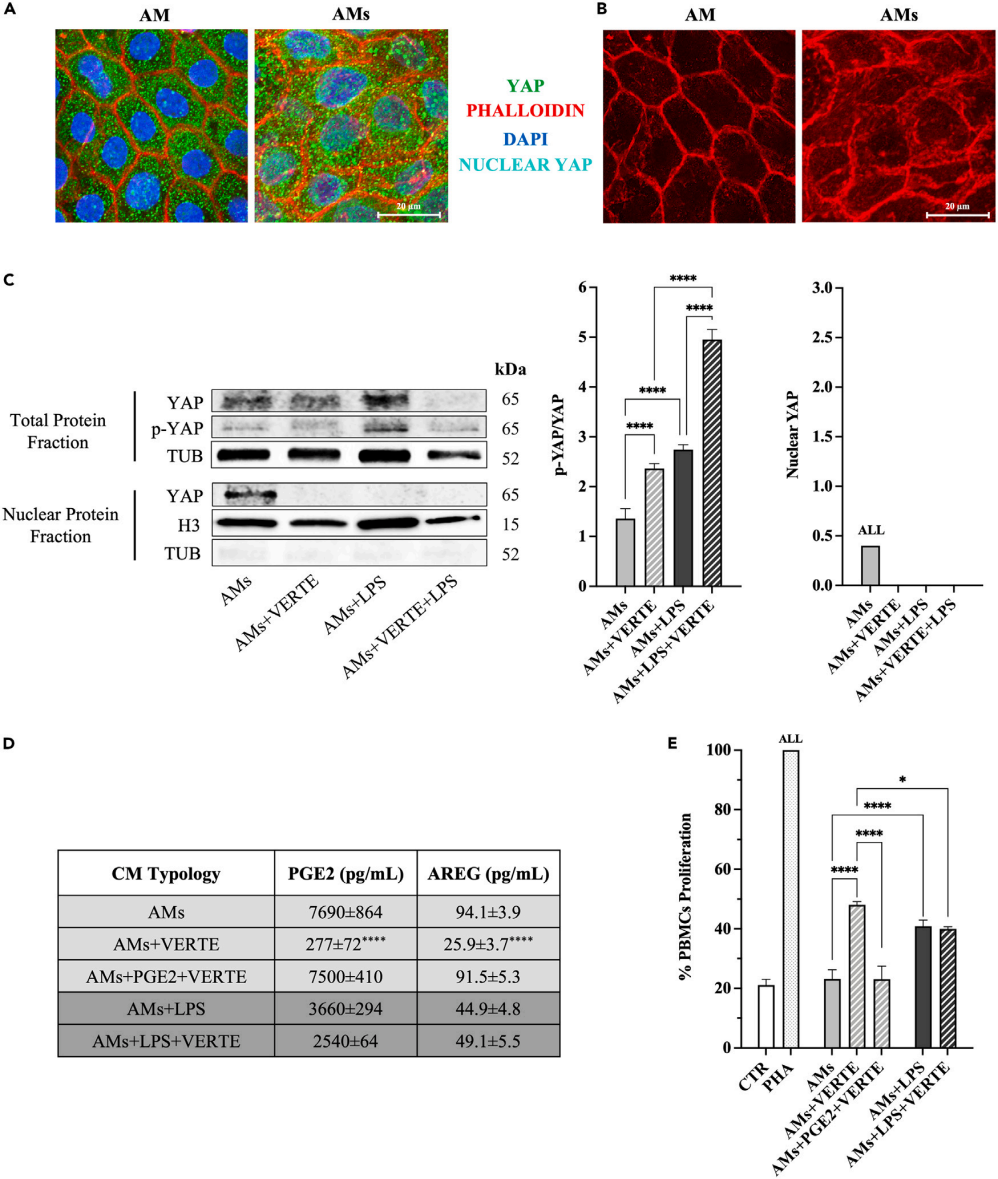
YAP nuclear translocation promoted by stretching mediated the enhanced release of PGE2 and AREG (A) Representative confocal images of co-immunofluorescence staining of YAP, phalloidin, and DAPI in amniotic-derived tissues (AM vs. AMs). (B) Confocal images showcasing sole phalloidin expression in representative samples of AM and AMs. (C) Representative WB images and relative densitometric analysis of p-YAP (normalized on YAP total content) and nuclear YAP protein expression relative to stretched amniotic-derived tissue±LPS cultured in the presence or absence of VERTE after tubulin normalization in each sample. YAP detected in total protein fraction was normalized to the corresponding housekeeping Tubulin expression, while nuclear YAP was normalized on histone H3. (D) PGE_2_ and AREG ELISA in the CM (±LPS) obtained from stretched amniotic-derived tissue cultured with VERTE. (E) CM (±LPS) obtained from stretched amniotic-derived tissue cultured with VERTE were tested on PHA-stimulated PBMCs (activation test after 48 h of culture). Data are shown as a ratio (%) to PHA-stimulated PBMCs. Data (mean ± SD) represent 3 independent sets of experiments (*n* = at least 3 biological replicates in each group per set; each biological replicate assayed in at least 3 technical replicates). All, ∗, and ∗∗∗∗ Statistically significant values between the different studied groups (*p* < 0.01, *p* < 0.05, and *p* < 0.0001, respectively). Scale bar, 20 μm.

The first experiments were designed to compare the expression and the subcellular location of YAP in native and stretched AM. Interestingly, YAP was constitutively expressed in AM at high concentrations, displaying an exclusive cytoplasmic distribution. Stretching induced a massive nuclear translocation of YAP in the epithelial layer (80% of epithelial cells; Figure 6A) whereas the intracellular protein distribution did not change in the underlying mesenchymal cells (data not shown). The mechano-transduction was accompanied, at the same time, by a conformational modification of actin. AM displayed an organized aggregation of actin filaments that with their cortical distribution reinforced the syncytial tissue organization of the epithelial layer. On the contrary, stretching promoted the re-organization of actin fibers which formed a dense, nest-like cell framework around the nucleus^61^^,^^62^^,^^63^ (Figure 6B).

Then, the functional role of the YAP was deepened in AMs by using verteporfin (VERTE), an inhibitor of the YAP-TEAD co-transcriptional interaction.^64^ In detail, the prompted YAP nuclear import induced by a mechanical stimulus was prevented in the absence of the Hippo pathway (AMs+VERTE). A similar YAP outcome was observed in LPS-treated AMs, where YAP did not undergo nuclear translocation. In fact, LPS in AMs induced a significant increase in protein phosphorylation, leading to YAP sequestration into the cytoplasm (Figure 6C). Of note, VERTE synergized with LPS by significantly increasing the levels of p-YAP (*p* < 0.0001 vs. AMs+LPS and AMs+VERTE; Figure 5C).

The role of YAP in mediating PGE_2_ and AREG release was also investigated. YAP inhibition induced a significant reduction of both PGE_2_ and AREG secretion (AMs vs. AMs+VERTE*; p* < 0.0001: Figure 6D). However, exogenous PGE_2_ supplementation (5,000 pg/mL) in AMs under VERTE influence reestablished AREG release (*p* < 0.0001 AMs+VERTE+PGE_2_ vs. AMs+VERTE and *p* > 0.05 AMs+VERTE+PGE_2_ vs. AMs; Figure 6D), thus demonstrating a YAP indirect influence on AREG transcription.

Therefore, the results confirm the key role of PGE_2_ in regulating AREG release in AMs, while also unveiling the role of YAP for the control of PGE_2_ transcription.

PBMC activation test confirmed this scenario. Indeed, the inhibitory influence of CM derived from AMs+VERTE was significantly reduced passing from 80% to 50% (AMs+VERTE vs. AMs, *p* < 0.0001; Figure 6E) and, of note, this effect was reverted by adding PGE_2_ (AMs+ PGE_2_+VERTE vs*.* AMs *p* > 0.05; Figure 6E). By contrast, VERTE had a very limited effect on PGE_2_ and AREG levels when combined with LPS stimulus. The CM of the latter experimental group exerted a similar immunosuppressive influence (AMs+LPS*+*VERTE vs. AMs+LPS, *p* > 0.05; Figure 6E). The results strengthen the evidence that LPS operates using a YAP-independent pathway in enhancing PGE_2_ secretion.

In conclusion, these findings reveal that the Hippo pathway is operative in the developed system and its effector, YAP, operates as a controller of PGE_2_ and AREG extracellular release.

### Stretching and LPS switch OFF/ON the Hippo pathway in amniotic-derived tissue

The role of Hippo pathway was finally confirmed by using the Rockefeller University large tumor suppressor (LATS) inhibitor (TRULI)^65^^,^^66^^,^^67^^,^^68^ to interfere with Hippo pathway by preventing cytoplasmic YAP phosphorylation.^66^^,^^67^^,^^68^^,^^69^^,^^70^^,^^71^^,^^72^

**Figure 7:**
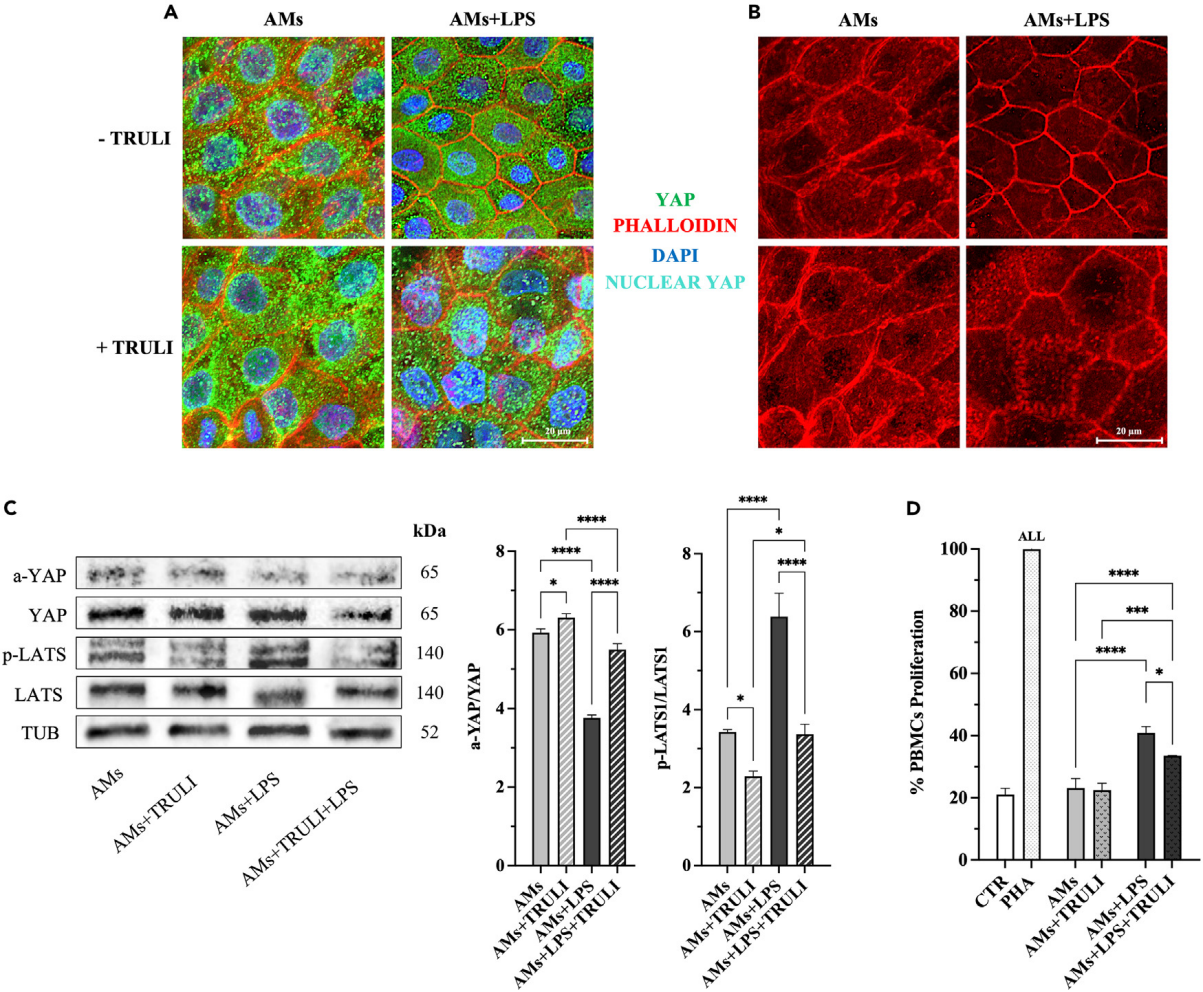
Stretching and LPS switch OFF/ ON the Hippo pathway in amniotic-derived tissue LATS-mediated alteration of Hippo signaling pathway in LPS stimulated AM/AMs. (A) Representative confocal images of co-immunofluorescence staining of YAP, phalloidin, and DAPI in stretched amniotic-derived tissue±LPS cultured in the presence or absence of TRULI. (B) Confocal images showcasing sole phalloidin expression in representative samples of AM and AMs±LPS and TRULI. (C) Representative WB images and relative densitometric analysis of a-YAP, YAP, p-LATS, and LATS protein expression relative to stretched amniotic-derived tissue±LPS cultured in the presence or absence of TRULI after tubulin normalization in each sample. (D) CM obtained from stretched amniotic-derived tissue±LPS cultured with TRULI were tested on PHA-stimulated PBMCs (activation test after 48 h of culture). Data (mean ± SD) represent 3 independent sets of experiments (*n* = at least 3 biological replicates in each group per set; each biological replicate assayed in at least 3 technical replicates). All, ∗, ∗∗∗, and ∗∗∗∗ Statistically significant values between the different studied groups (*p* < 0.01, *p* < 0.05, *p* < 0.001, and *p* < 0.0001, respectively). Scale bar, 20 μm.

Confocal immunofluorescence analysis of YAP in AMs with/without LPS strongly validated the dynamic modulation of the Hippo pathway. (Figure 7A). More in detail, LPS input prevented the nuclear import of YAP induced by stretching (Figures 6A and 7A). At the same time, LPS countered the conformational change of actin filaments induced by stretching^62^ (Figures 6B and 7B), re-establishing the native AM cortical distribution of the cytoskeleton proteins^73^ (Figure 7B). TRULI abolished the effect of LPS on AMs by allowing the nuclear import of YAP (Figure 7A). At the same time, TRULI also affected actin remodeling inducing a stretching nest-like distribution^61^ (Figure 7B).

The effectiveness of TRULI in inhibiting LATS in AMs with/without LPS was confirmed through WB (Figure 7C). TRULI effectively inhibited the activation of LATS, either under basal conditions or stimulation with LPS (*p* < 0.05 and *p* < 0.0001 vs. without TRULI, respectively; Figure 7C). Of note, TRULI was also effective in significantly increasing the expression of active YAP (a-YAP) in the AM+LPS condition (*p* < 0.05 vs. without TRULI; Figure 7C).

Both confocal and WB analysis confirmed the role of LPS in activating the Hippo pathway. Indeed, it promoted LATS activity (*p* < 0.0001 vs. AMs; Figure 7C), and restricted YAP into the cytoplasm (Figure 7A), thus inhibiting its activation (*p* < 0.05 vs. AMs; Figure 7C).

Finally, the functional influence of LATS was assessed on the immunomodulatory paracrine influence of AMs by demonstrating that TRULI antagonized LPS stimulus. Indeed, TRULI significantly enhanced the immunosuppressive potential of CM derived from AMs after LPS exposition (*p* < 0.05, AMs+LPS+TRULI vs. AMs+LPS; Figure 7D) by significantly increasing AREG content (*p* < 0.05: data not shown). Conversely, no immunosuppressive differences were observed in AMs exposed to TRULI treatment (*p >* 0.05 vs. AMs; Figure 7D), probably resulting from the previously inhibitory state of LATS induced by stretching.

In conclusion, the LPS signal engages the Hippo pathway activating LATS function in AMs. LATS is responsible for the phosphorylation of YAP.^74^^,^^75^ The activation of the Hippo pathway prevents YAP nuclear import induced by stretching, avoiding the transcriptional advantage of mechanical input on PGE_2_ secretion.

## Discussion

The amniotic membrane holds significant clinical appeal, as it is not only integral to sustaining pregnancy progress but also serves as a valuable biological source for fetal stem cells.^3^

Amniotic cells/membrane are recognized to express a widespread immunomodulatory action by controlling immune cells under both physiological and applicative conditions.^13^ Different studies, indeed, have shown that these cells/tissue strongly suppress T cell proliferation induced by allogeneic stimuli *in vitro*,^76^^,^^77^ receptor cross-linking (anti-CD3), mitogens such as concanavalin A and PHA, or by recall antigen.^18^^,^^22^^,^^78^^,^^79^ Interestingly, amniotic cells can also suppress the proliferation of healthy PBMCs or those isolated from patients with rheumatoid arthritis.^80^

However, even if the paracrine-mediated crosstalk between amnion-derived cells/membrane and immune cells has been well documented *in vitro* and *in vivo* for its biological outcomes*,* the underlying molecular basis is only partially defined. PGE_2_, identified as a pivotal immunomodulatory agent,^48^^,^^81^^,^^82^^,^^83^ has demonstrated its anti-proliferative effects and also as a bioactive lipidic component of term amniotic derivatives.^18^^,^^84^ Specifically, placental-derived PGE_2_, acting through the EP4 receptor, was shown to be a key effector in mediating the inhibition of T cell proliferation.^18^^,^^84^ Taken together, these findings strongly suggest a direct involvement of PGE_2_ released from amniotic membranes in inhibiting the proliferation of immune cells.^18^^,^^84^ However, in the current investigation, involving pre-term amniotic cells/membrane, maintained under a controlled cultural condition to preserve the native epithelial phenotype, PGE_2_ appeared to function more as a behind-the-scenes effector, exerting its influence through the release of AREG. Indeed, the secretion of PGE_2_ from AEC and AM, either under basal or stimulating conditions (LPS and stretching), has never been adequate to express a direct control over PBMCs proliferation and/or apoptosis. On the contrary, the obtained results unequivocally establish the indirect role of PGE_2_ in amniotic-derived cells/tissue immunosuppressive activity by coordinating AREG release. The emerged evidence supports AREG’s immunosuppressive pivotal role, targeting PBMCs activity, intracellular NFAT signaling in Jurkat cells, as well as immune cell recruitment in an acute inflammatory model performed *in vivo* on zebrafish larvae. A similar crosstalk between PGE_2_ e AREG has been well documented in cancer, where new therapeutic strategies targeting these signaling pathways have been developed.^49^ The interplay between the PGE_2_ cascade and EGFR activating pro-tumoral mechanisms recognized two main pathways. Depending on the cell model, PGE_2_ may affect EGFR activity through the mobilization of intracellular signaling pathways, which promotes transactivation and phosphorylation of EGFR,^50^^,^^51^^,^^52^^,^^53^^,^^85^ or PGE_2_-induced extracellular mobilization of EGFR ligands like in AEC/AM.^54^^,^^55^^,^^56^ For instance, Oshima and co-workers demonstrated that, in mouse models of gastric cancer, the expression levels of EGFR ligands and MMPs increase in a PGE_2_-pathway-dependent manner.^54^^,^^55^ Indeed, PGE_2_ impacted the release of AREG also by upregulating MMPs thus activating EGFR via EP4 receptor.^54^^,^^55^

**Figure 8:**
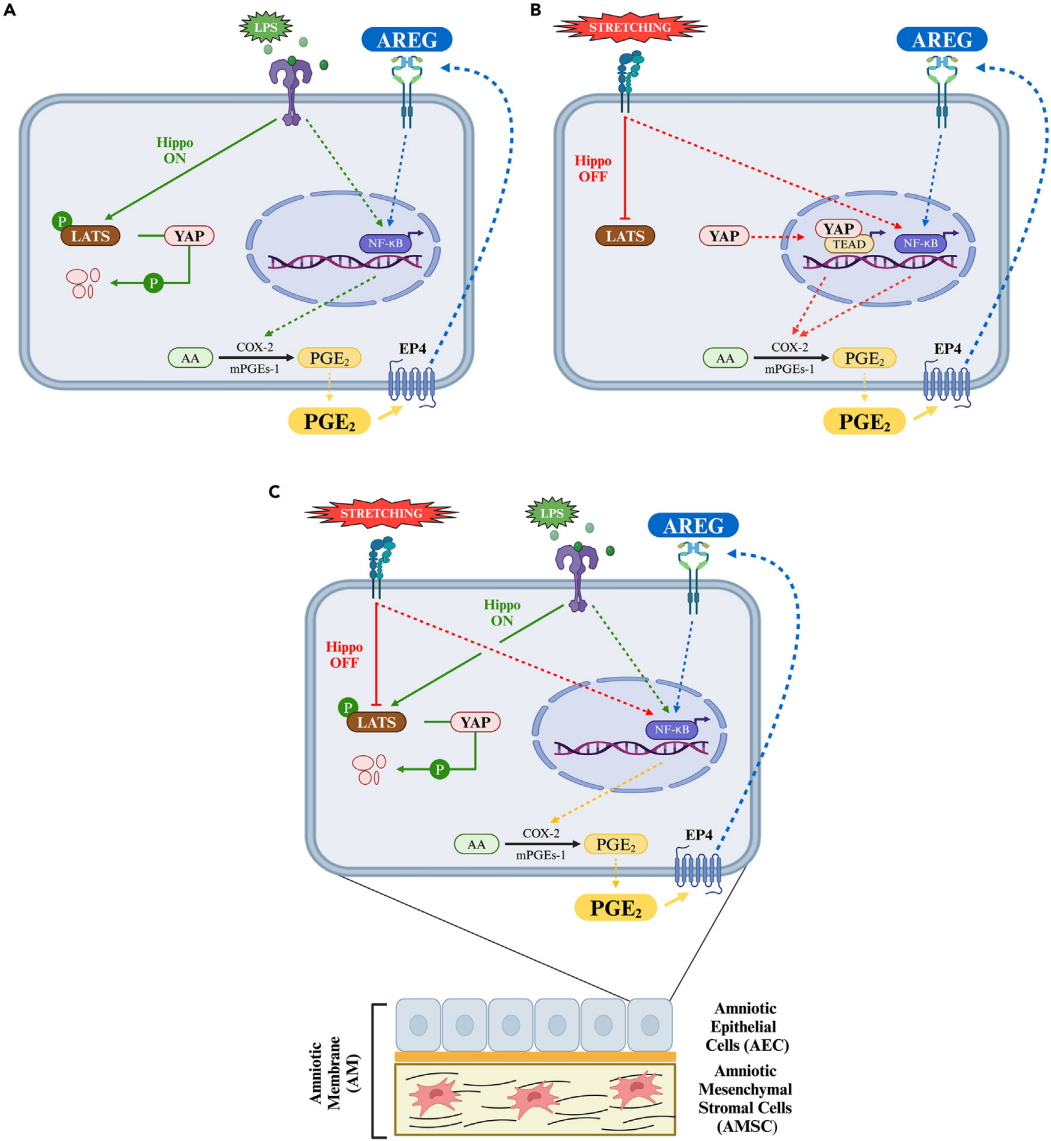
Schematic of the mechanisms involved in amniotic-derived cells/tissue’ immunomodulatory properties (A–C) Schematic of the proposed mechanism outlining how amniotic-derived cells/tissue respond to (A) LPS, (B) stretching, and (C) a combination of both, leading to the release of PGE_2_ and AREG.

On the contrary, although AREG has been described in several reproductive tissues as a modulator of key reproduction events,^30^^,^^86^^,^^87^ the EGFR ligand had never been documented in amniotic tissues. Here, the COX2/PGE_2_/EP4 inflammatory axis discovered for the modulation of EGFR signaling was demonstrated to be operative physiologically in driving AM/AEC native paracrine activity as well as their response to external stimuli. Matching the literature and the present results, it can be hypothesized a general model of PGE_2_ and AREG release in amniotic-derived cells/tissues. LPS triggers the validated nuclear factor kappa-light-chain-enhancer of activated B cells (NF-kB) pathway, leading to PGE_2_ production, and subsequently, to AREG release via COX-2/PGE_2_/EP4 axis^29^^,^^74^^,^^88^ (Figure 8A). This process strengthens the system by establishing a positive feedback loop on NF-kB activation via AREG/EGFR axis.^74^^,^^89^^,^^90^ For instance, Heo et al. highlighted the role of AREG in influencing the expression of COX-2 through the NF-κB signaling pathway.^91^ Additionally, LPS activates the canonical regulatory Hippo pathway leading to YAP degradation through LATS activation.^74^^,^^75^ On the other hand, stretching input (Figure 8B) switches the Hippo pathway “OFF” by inhibiting LATS and promoting the nuclear translocation of YAP.^75^^,^^92^^,^^93^ This cascade of events alternatively activates a feedforward PGE_2_ signaling loop mediated by the YAP-TEAD transcriptional mechanism upregulating microsomal prostaglandin E synthase-1 (mPGEs-1).^94^ As a direct consequence, AREG release significantly increases taking advantage of both the transcriptional paths increasing PGE_2_ (NF-kB and YAP-TEAD).^29^^,^^88^^,^^89^^,^^90^^,^^95^^,^^96^ Of note, stretching did not influence the underlying amniotic membrane mesenchymal layer. The answer to the different contributions of the two amniotic layers lies in the unique nature of epithelial cells, which are more sensitive to mechanical stimuli than mesenchymal ones, owning several types of cell-to-cell junctions, and tight junctions, which are known to play important roles in maintaining the epithelial phenotype. Recent studies shed light on the role of tricellular junctions (TCJs) and adherens junctions in sensing and responding to mechanical forces, respectively.^97^^,^^98^ These junctions play a crucial role in coordinating cell behavior by sensing differences in cytoskeletal geometry, which is essential for tissue homeostasis.^99^ Moreover, Yap et al. underscore the importance of force generation and sensing at cell-to-cell junctions, particularly through cadherins.^100^ Consequently, during epithelial-mesenchymal transition (EMT), marked by the loss of epithelial markers, including E-cadherin and tight junction proteins, cells experience a diminished sensitivity to mechanical stimuli.^101^^,^^102^ Finally, the simultaneous exposure to stretching and LPS inputs (Figure 8C) promotes concurrently the inhibition and activation of the Hippo pathway.^74^^,^^75^^,^^92^ However, LPS stimulus prevails leading to YAP degradation and suppressing the stretching effect. Additionally, this combined exposure adversely impacts the conformational change of actin filaments induced by stretching, leading to a cortical distribution of cytoskeleton proteins.^62^

### Limitations of the study

Additional aspects should be considered to move toward the targeted handling of such mechanisms to develop innovative amniotic stem cell free-based immune protocols. For instance, in this study, stretching emerges as a crucial factor to influence the potential immunomodulatory effects of amniotic cells/tissue. Indeed, this stimulus, mediated by mechanotransduction processes, plays a pivotal physiological role in various aspects of pregnancy and fetal development up to labor.^103^^,^^104^ It is indispensable for supporting fetal growth and the delivery, impacting processes like inflammation, apoptosis, and extracellular matrix remodeling.^103^^,^^104^ However, not all mechanisms underlying this process have been fully investigated, including the interplay between NF-kB and mechanotransduction. Mohan et al.'s research focused on the impact of mechanical stretch on COX2 expression and the activity of activator protein-1 (AP1) and NF-kB in human amnion cells.^103^ They demonstrated that in pre-term labor, NF-κB and AP-1 could be activated by cytokines, stretch, or bacterial endotoxins via Toll-like receptors or a combination of all the three, leading to increased PGE_2_ synthesis via COX-2.^103^ This supports the present study, reinforcing the concept of stretching as a more potent stimulus than LPS, activating two distinct mechanisms of PGE_2_ and AREG production through YAP and NF-κB, which amplified through a loop via AREG/EGFR. Moreover, the precise structural motif of AREG remains to be defined to fully grasp the signaling potential used by AEC/AM and their derivatives.^105^ Furthermore, although AREG and other EGF family members are originally described as epithelial cell-derived factors, several evidence shows that EGFR ligands can be expressed by multiple populations of activated immune cells in a variety of inflammatory conditions. Moreover, in other districts, AREG plays its local action by generating a bi-directional crosstalk between immune and epithelial cells orchestrating simultaneously immunity, inflammation, and tissue repair to enhance host-protective response.^35^ As previously seen in other cellular systems, AREG exert its immunomodulatory activity on PBMCs and Jurkat also through the activation of latent TGF-β, converting it into its bioactive form. This activation process involves integrin-αV-containing complexes, which facilitate the local release of TGF-β.^57^ Furthermore, TGF-β signaling is known to involve integrins such as αVβ8 and αVβ6, which are crucial for the activation of latent TGF-β complexes. This mechanism has been shown to be essential in maintaining immune homeostasis and preventing inflammatory diseases.^106^

Addressing these gaps in knowledge in AEC, the present results have the merit to demonstrate the strong association between the COX-2/PGE_2_/EP4 pathway and AREG/EGFR axis by discovering a key molecular immunomodulator.

In conclusion, this comprehensive model sheds light on the interplay among the intracellular signaling pathways governing immunomodulatory actions of amniotic-derived cells/tissue. Moreover, the obtained findings not only provide a valuable guidance to develop targeted therapeutic interventions but also underscore the necessity of further investigations to enhance our understanding of these intricate biological processes and their crosstalk with the immune cell counterpart.

## STAR★Methods

### Key resources table

**Table:** 

REAGENT or RESOURCE	SOURCE	IDENTIFIER
**Antibodies**		

Mouse monoclonal anti-CD3	Invitrogen, Thermo Fisher Scientific	Cat# 16-0037-81; RRID: AB_468854
Mouse monoclonal anti-CD28	Invitrogen, Thermo Fisher Scientific	Cat# 16-0289-81; RRID: AB_468926
Goat polyclonal anti-AREG	R&D Systems	Cat# AF262; RRID: AB_2243124
Rabbit monoclonal anti-YAP for IHC	Sigma-Aldrich	Cat# SAB2108066
Mouse monoclonal Anti-rabbit Alexa Fluor 488	Molecular Probes	Cat# AB150077; RRID: AB_2630356
Rabbit monoclonal anti-p-SMAD2	Cell Signaling Technology	Cat# 3108; RRID: AB_490941
Rabbit polyclonal anti-SMAD2/3	Cell Signaling Technology	Cat# 3102; RRID: AB_10698742
Rabbit polyclonal anti-p-FAK	Bioss Inc.	Cat# bs-3164R; RRID: AB_10856600
Sheep polyclonal anti-FAK	R&D Systems, Inc.	Cat# AF4467; RRID: AB_2237882
Rabbit polyclonal anti-β-Actin	Santa Cruz Biotechnology	Cat# SC7210; RRID: AB_2223518
Goat polyclonal anti-COX2	Abcam Limited	Cat# AB23672; RRID: AB_731725
Rabbit polyclonal anti-PGE2S	Cayman Chemical Company	Cat# 160145; RRID: AB_10894462
Rabbit monoclonal anti-YAP	Cell Signaling Technology	Cat# 14074; RRID: AB_2650491
Rabbit polyclonal anti-p-YAP	Invitrogen, Thermo Fisher Scientific	Cat# PA5-121279; RRID: AB_2914851
Rabbit monoclonal anti-a-YAP	Cell Signaling Technology	Cat# 29495; RRID: AB_2798974
Rabbit monoclonal anti-LATS	Cell Signaling Technology	Cat# 3477; RRID: AB_2133513
Rabbit polyclonal anti-p-LATS	Cell Signaling Technology	Cat# 9157; RRID: AB_2133515
Mouse monoclonal anti-Tubulin	Cell Signaling Technology	Cat# 3873; RRID: AB_1904178
Rabbit monoclonal anti-H3	PTM Biolabs Inc.	Cat# PTM-1001RM

**Chemicals, peptides, and recombinant proteins**		

LPS	Sigma-Aldrich	Cat# L2637
PHA-L	Invitrogen	Cat# 00-4977-93
Penicillin-Streptomycin	Lonza	Cat# DE 17-602E
Amphotericin B	Euroclone	Cat# ECM0009D
L-Glutamine	Euroclone	Cat# ECB300D
P4	Sigma-Aldrich	Cat# P8783
Ficoll-Paque PLUS	Cytiva	Cat# GE17-1440-02
Fluoromount	Sigma-Aldrich	Cat# F4680
DAPI	Sigma-Aldrich	Cat# D9542
Formol 4%	VWR	Cat# 11699404
Triton X-100	Sigma -Aldrich	Cat# T8787
PBS	Sigma-Aldrich	Cat# P3813
PBS with Ca^+2^ and Mg^+^	Sigma-Aldrich	Cat# D8662
BSA	Sigma-Aldrich	Cat# P3813
Quick Start™ Bradford 1x Dye Reagent	Bio-Rad Laboratories	Cat# 5000205
4X Laemmli Sample buffer	Bio-Rad Laboratories	Cat# 1610747
Phosphatase Inhibitor	SERVA Electrophoresis GmbH	Cat# 39055
Protease Inhibitor Cocktails	Sigma-Aldrich	Cat# P2714
Precast gel with a density gradient of 4-15%	Bio-Rad Laboratories	Cat# 4568083
Nitrocellulose membranes	Bio-Rad Laboratories	Cat# 1620145
Trans-Blot Turbo 5x Transfer Buffer	Bio-Rad Laboratories	Cat# 10026938
Every blot blocking solution	Bio-Rad Laboratories	Cat# 12010020
1X TBS 1% Casein Blocker	Bio-Rad Laboratories	Cat# 1610782
ClarityMax ECL reagent	Bio-Rad Laboratories	Cat# 1705062
Trypsin-EDTA 0.25%	Sigma-Aldrich	Cat# T4049
Normocin™	InvivoGen	Cat#ANT-NR-2
Blasticidin	InvivoGen	Cat#ANT-BL-1
Zeocin®	InvivoGen	Cat#ANT-ZN-1
Indomethacin	Sigma-Aldrich	Cat# S-17378
L-161,982	Cayman Chemical	Cat# AB120947
SB-505124	MedChemExpress	Cat# HY-13521
Verteporfin	Sigma-Aldrich	Cat# SML0534
TRULI	Selleck Chemicals	Cat# E1061
[methyl-3H] thymidine	Perkin Elmer/New England Nuclear Corporation	Cat# NET355001MC
DMSO	Sigma-Aldrich	Cat# D8418
QUANTI-luc™, Luciferase Detection Reagent	InvivoGen	Cat# REP-QLC4LG1
Agarose A/G resin	Merck Millipore	Cat# AF262
TRICAINE PHARMAQ 1000 mg/g	PHARMAQ AS	Cat# 104497045
Tween 20	CARLO ERBA Reagents S.r.l.	Cat# 600481
Phalloidin TRITC	Sigma-Aldrich	Cat# P1941
TGF-β1	PeproTech	Cat# 100-21-50UG

**Critical commercial assays**		

CellTiter96 Aqueous One Solution Cell Proliferation Assay	Promega	Cat# G3582
Click-iT™	Invitrogen, Thermo Fisher Scientific	Cat# C10499
RealTime-Glo™ Annexin V apoptosis assay	Promega	Cat# JA1011
Prostaglandin E2 Human ELISA Kit	Thermo Fisher Scientific	Cat# KHL1701
Sheep Amphiregulin (AREG) ELISA kit	MyBiosource	Cat# MBS044705
NE-PER™ Nuclear and Cytoplasmic Extraction Reagents	Thermo Fisher Scientific	Cat# 78835

**Experimental models: Cell lines**		

AEC	University of Teramo	N/A
PBMCs	University of Teramo	N/A
Jurkat-Lucia™ NFAT cells	InvivoGen	Cat# jktl-nfat-cd28

**Experimental models: Organisms/strains**		

Zebrafish transgenic larvae (*Tg(lysC:DsRed2*)	University of Teramo	N/A

**Software and algorithms**		

Image J 1.53k	NIH	https://imagej.nih.gov/ij/
GraphPad Prism 10	Graphpad	https://www.graphpad.com
Biorender	Biorender	https://www.biorender.com

**Other**		

FBS	Gibco	Cat# 10270106
α MEM	Euroclone	Cat# ECM0850L
IMDM	Thermo Fisher Scientific	Cat# 12440053

### Resource availability

#### Lead contact

Further information should be directed and will be fulfilled by the lead contact, Dr. Giuseppe Prencipe (gprencipe@unite.it).

#### Materials availability

This study did not generate new unique reagents or materials.

#### Data and code availability

• All data reported in this paper will be shared by the lead contact upon request.
• This paper does not report original code.
• Any additional information required to reanalyze the data reported in this work paper is available from the lead contact upon request.

### Experimental model and study participant details

#### Ethical statement

The amniotic cells/tissues used for the present research have been isolated from adult pregnant sheep. No authorization is needed for the collection and use of such tissues/cells since amniotic membranes are waste products of animals slaughtered for food consumption.

The zebrafish developmental stage at 72 hpf did not fall into the regulatory frameworks dealing with animal experimentation, and all the experiments complied with “Directive 2010/63/EU of the European Parliament and of the Council of 22 September 2010 on the protection of animals used for scientific purposes” and with the Italian law “D.Lgs n. 26 4 marzo 2014 Attuazione della direttiva 2010/63/UE sulla protezione degli animali utilizzati a fini scientifici”.

#### Isolation and culture of ovine amniotic-derived AEC/AM

AEC were isolated from AM isolated from pregnant Appenninica breed sheep at middle stage of gestation (25-30 cm in fetus length),^24^ to avoid the activation of epithelial-mesenchymal transition (EMT) at term stage.^24^^,^^107^^,^^108^ Briefly, the uterus wall was carefully opened to isolate sterile amnios. Then, AM was mechanically peeled from the chorion with the aid of a stereomicroscope, and the tissue was dissected into 2–3 cm fragments. The AM pieces were washed in phosphate-buffered saline (Ca^+2^, Mg^+2^ free) (PBS; #P3813; Sigma-Aldrich, St. Louis, MO, USA) before isolating AEC by incubating them under gentle agitation in 0.25% Trypsin-EDTA (#T4049; Sigma-Aldrich, St. Louis, MO, USA) at 38.5°C for 40 min. The enzymatic digestion was blocked by adding 10% Fetal Bovine Serum (FBS; #10270106; Gibco, Thermo Fisher Scientific, Waltham, MA, USA) before filtration on a 40 μm pore membrane. The isolated cells were pelleted by centrifugation at 500g for 10 minutes before counting the vital ones using LUNA-II™ Automated Cell Counter (Logos Biosystems Inc., Gyeonggi-do, Korea) with the aid of trypan blue staining.

AEC were subsequently seeded at 10^4^ cells/well on 6 well plates using α-Minimum Essential Medium Eagle (α-MEM; #ECM0850L; Euroclone S.p.A., Milan, Italy), supplemented with 10% inactivated FBS (#10270106; Gibco, Thermo Fisher Scientific, Waltham, MA, USA), 1% L-Glutamine (#ECB300D; Euroclone S.p.A., Milan, Italy), 1% amphotericin B (#EUM0009D; Euroclone S.p.A., Milan, Italy), and 1% penicillin/streptomycin (#DE17-602E; Lonza, Basel, Switzerland) (hereafter referred as a complete medium), with 25 μM Progesterone (P_4_) (4 pregnene-3,20-dione, P_4_, #P8783; Sigma-Aldrich Corp., St. Louis, MI, USA) to prevent Epithelial-to Mesenchymal Transition (EMT).^109^^,^^110^ The cells were maintained at 38°C with 5% CO_2_ upon 70% confluence had been reached. Then, AEC were trypsinized (0.05% Trypsin-EDTA) for further experimental procedures.

AM was isolated from the fetus's placenta as described above. Following extraction, the membranes underwent thorough washing and were maintained in PBS with Ca^+2^ and Mg^+^ (#D8662; Sigma-Aldrich, St. Louis, MO, USA). The AM pieces were precisely cut into 1,4x1,4 cm dimensions (approximately 2 cm^2^). Subsequently, to obtain the stretched condition (AMs), 2 x 2 cm AM pieces were positioned between two 3D-printed round supports, simulating a mild radial tension mimicking that exerted from amniotic fluid during the gestation.^111^^,^^112^^,^^113^ To ensure a perfect fit of the AM pieces to the support structures, any excess tissue was trimmed (approximately 2 cm^2^ remaining). The cell concentration on the resulting 2 cm^2^ AM and AMs pieces was comparable to that achieved with 70% confluence in cells cultured in 6-well plates. Subsequently, all samples underwent incubation with complete media enriched with 25 μM P_4_ at 38.5°C in a 5% CO2 environment.

#### Isolation of ovine PBMCs

Ovine PBMCs were isolated through a density gradient centrifugation starting from 16 mL of fresh peripheral blood collected at the slaughterhouse. The gradient was built up with 12 mL Ficoll-Paque PLUS (#GE17-1440-02; Cytiva, Marlborough, MA, USA) following the manufacturer’s instructions. Following the procedures mentioned above, the cells were preserved by storing them in liquid nitrogen until needed for immunological tests.

#### Isolation of human PBMCs

Healthy donor buffy coats were purchased from the University Clinic for Blood Group Serology and Transfusion Medicine in Vienna, Austria, and the Austrian Red Cross. PBMCs were isolated through standard density gradient centrifugation using Ficoll-Paque PLUS (#GE17-1440-02; Marlborough, MA, USA). Following the procedures mentioned above, the cells were preserved by storing them in liquid nitrogen until needed for immunological tests.

#### Culture of Jurkat reporter cells

Jurkat-Lucia™ NFAT cells (#jktl-nfat-cd28; InvivoGen, Toulouse, France), are a sensitive report T cell line that enables the measurement of NFAT activity via an NFAT-inducible Lucia luciferase construct. Cells were cultured in Iscove's Modified Dulbecco's Medium (IMDM; #12440053; Gibco, Thermo Fisher Scientific, Waltham, MA, USA) supplemented with 10% heat-inactivated FBS (#10270-106; Gibco, Thermo Fisher Scientific, Waltham, MA, USA), 100 μg/mL penicillin/streptomycin (#DE17-602E; Lonza, Basel, Switzerland), 100 μg/mL Normocin™ (#ANT-NR-2; InvivoGen, Toulouse, France), 5 μg/mL Blasticidin (#ANT-BL-1; InvivoGen, Toulouse, France) and 100 μg/mL Zeocin® (#ANT-ZN-1; InvivoGen, Toulouse, France) and passaged every 2 days until the 3^rd^ passage to maintain the cellular density within the suggested threshold at 37°C under 5% CO2 atmosphere.

#### Zebrafish

The zebrafish transgenic line *Tg(lysC:DsRed2*) used in the experiments was obtained from the University of Teramo facility (protocol number n. 4236). Adult fish were kept in a circulating system tank (Tecniplast S.p.a., Buguggiate, Italy). The tank temperature was generally maintained between 27°C-28°C, the pH at 7±0.2, the conductivity at 500±100 μS/cm, and the lighting conditions in the room were 14:10h (light: dark). Animals were fed twice a day with live food (*Artemia salina*) and supplemented with ZEBRAFEED (Sparos, Olhão, Portugal). Zebrafish eggs were collected from timed pairwise spawning using sloping breeding tanks (Tecniplast S.p.a., Buguggiate, Italy). Immediately after spawning, which was initiated by morning light, the eggs were collected, rinsed and unfertilized eggs or injured embryos were eliminated. The embryos were maintained at 27°C in a 100mm glass etri dish in dilution water (DW) until the experimental time (72 hpf).

### Method details

#### CM production from amniotic-derived AEC/AM

CM production was started once freshly isolated AEC reached 70% confluence on a 6-well plate. Then, the cells were preconditioned in serum-free medium (αMEM + 1% penicillin/streptomycin + 1% Amphotericin B + 1% L-Glutamine) for 4h. According to previous studies in other cellular models^114^ and AEC,^110^^,^^115^ some of them were exposed to the inflammatory LPS stimulus (1 μg/ml, #L2637; Sigma-Aldrich, St. Louis, MO, USA) for 1h. Afterward, all the AEC groups (CTR and LPS) were washed twice with serum-free medium and cultured in the same for 24h before CM collection. Similarly, AM was incubated in serum-free medium supplemented with P_4_ in 2D (AM: on the bottom of the well) and 3D (AMs: stretched on holder) conditions for 4h. Afterward, some AMs were exposed to LPS (1 μg/ml) for 1h. All AM groups were washed 2 times in serum-free medium before the 24h incubation. Subsequently, the AEC and AM-derived CM were centrifuged at 500g for 10 minutes and the supernatants were stored at -80°C.

To deepen the intracellular pathways involved in mediating the immunosuppressive properties of amniotic derivates (AEC and AM CM), the following treatments were performed:

• inhibition of PGE_2_ synthesis using 10 μM of indomethacin (#S-17378; Sigma-Aldrich, St. Louis, MO, USA) during the 24h-serum free medium incubation.
• inhibition of EP4 receptor using 10 μM of L-161,982 during the 24h-serum free medium incubation (#AB120947; Cayman Chemical, Ann Arbor, MI).
• inhibition of TGF-β using 2 μM of SB-505124 during the 24h-serum free medium incubation (#HY-13521; MedChemExpress, NJ, USA).
• inhibition of YAP nuclear translocation by exposing AMs to 10 μM of Verteporfin (#SML0534; Sigma-Aldrich, St. Louis, MO, USA) during the 24h-serum free medium incubation.
• inhibition of LATS, using on AMs 10 μM of TRULI (#E1061; Selleck Chemicals, Houston, Texas, USA) during 24h-serum free medium incubation carried out with or without LPS.

#### PBMCs activation test

The immunomodulatory effect of different CM (AEC, AM, and AMs ±LPS) was verified using the MTS PBMCs activation test. In detail, PBMCs were seeded in 96-well plates at the concentration of 2 × 10^5^ cells, activated with 10 μg/mL of phytohemagglutinin (PHA-L; #00-4977-93; Invitrogen, Thermo Fisher Scientific, Waltham, MA, USA) and cultured for 48h with or without CM. Then, PBMCs proliferation was assessed by using the “CellTiter 96 Aqueous One Solution Cell Proliferation Assay” (#G3582; Promega, Corporation, Madison, WI, USA) following the manufacturer’s instruction protocol. Data were normalized on PHA-activated PBMCs.

#### DNA synthesis assessment

To evaluate the inhibitory effects of CM on ovine PBMCs’ DNA synthesis, the EdU kit (#C10499; Invitrogen, Thermo Fisher Scientific, Waltham, MA, USA) was employed following the manufacturer’s instructions. Specifically, PBMCs were seeded in 96-well plates at the concentration of 2 × 10^5^ cells, activated with 10 μg/mL of phytohemagglutinin (PHA; #00-4977-93; Invitrogen, Thermo Fisher Scientific, Waltham, MA, USA), and cultured for 48h with or without CM Data were normalized on PHA-activated PBMCs.

In addition, the inhibitory influence of CM on PBMCs’ DNA synthesis, was alternatively tested on human PBMCs using a more sensible radio-labeling assay. In detail, human PBMCs were seeded in 96-well plates at the concentration of 1 × 10^5^ cells, activated via CD3 (partial activation) or CD3/CD28 (complete activation) (2 μg/mL; #16-0037-81 and #16-0289-81, respectively; Invitrogen, Thermo Fisher Scientific, Waltham, MA, USA) coated high binding plate (#9018; Corning, NY, USA), and cultured for 48h with CM derived from the various AEC, AM and AMs groups. Subsequently, cells were labeled with 0.05 mCi/well of [methyl-3H] thymidine (#NET355001MC; Perkin Elmer/New England Nuclear Corporation, Wellesley, MA), and cultured for a further 18h before harvesting. DNA synthesis analysis was performed on a microplate scintillation counter (Topcount; Packard, Meriden, CT).

#### Cell death and early apoptosis assay

To verify if the inhibitory effect of CM on ovine PHA-activated PBMCs was dependent on cell death, the RealTime-Glo Annexin V apoptosis assay (#JA1011; Promega, Corporation, Madison, WI, USA) was performed, following the manufacturer’s instructions. PBMCs treated with 10% v/v of DMSO (#D8418; Sigma-Aldrich, St. Louis, MO, USA) were taken as a positive control of apoptosis. Luminescence was assessed by using EnSpire Multimode Plate Reader (PerkinElmer, Waltham, MA, USA). Data were normalized on PHA-activated PBMCs.

#### NFAT pathway activation

To test CM’ effects on specific intracellular immune cell signaling pathways controlling proliferation, Jurkat-Lucia™ NFAT cells were used. Reporter Jurkat cells amplified for 3 passages were seeded at a concentration of 3 × 10^5^ cells on a 96-well high binding plate (#9018; Corning, NY, USA), previously coated with a CD3/CD28 (2 μg/mL, #16-0037-81 and #16-0289-81, respectively; Invitrogen, Thermo Fisher Scientific, Waltham, MA, USA). The culture was maintained for 48h in the presence or absence of CM. The evaluation of the mean luminescence value was assessed, after the supplement of the QUANTI-luc™, Luciferase Detection Reagent (#REP-QLC4LG1; InvivoGen, Toulouse, France), by using EnSpire® Multimode Plate Reader (PerkinElmer, Waltham, MA, USA). Data were normalized on the CD3/CD28 stimulated Jurkat.

#### ELISA assays

The CM were assayed for PGE_2_ and AREG concentration using the “Prostaglandin E2 Human ELISA Kit” (#KHL1701; Invitrogen, Thermo Fisher Scientific, Waltham, MA, USA) and “Sheep Amphiregulin (AREG) ELISA kit” (#MBS044705; MyBiosource, Southern California, San Diego, USA), respectively. The assays were performed according to the manufacturer’s instructions.

#### PGE_2_ and AREG dose-effect response on PBMCs

PBMCs activation test was also employed to verify PGE_2_ and/or AREG influence on the immune cells by designing dose-dependent experiments by reproducing the concentrations of the molecules detected into the CM: from 0 to 10.000 pg/mL for PGE_2_ and, from 0 to 200 pg/mL for AREG. The same protocol was used to confirm that the PGE_2_ effect was mediated by the EP4 receptor, while the CM one by AREG adopting the specific inhibitor or an immunoprecipitation approach, respectively.

#### Immunoprecipitation of AREG

AREG was removed from the CM by performing an immunoprecipitation protocol. The samples were incubated with the AREG primary antibody (1:200, #AF262; R&D Systems, Inc., MN, USA) overnight at 4°C in rotation. Then, 50 μL of the Agarose A/G resin (#IP05; Merck Millipore, Burlington, MA, USA) was added to the samples and incubated for 1.5h at 4°C in rotation. After the incubation period, the samples were centrifuged at 14.000 g for 1 minute to pellet the agarose. Afterward, AREG-free CM (supernatant) was collected. The efficiency of the immunoprecipitation assay was also verified by testing AREG soluble concentration.

#### CM’ immunomodulatory properties on transgenic zebrafish

To assess the effects of CM *in vivo*, *Tg(lysC:DsRed2)* zebrafish larvae at 72 hpf were used. Firstly, the larvae were anesthetized with TRICAINE PHARMAQ (PHARMAQ AS, Norway) at 0.02% and with a sharp scalpel, the distal tip of the tailfin was dissected to induce acute inflammation. Then, the larvae were exposed to CM derived from AEC and AMs (1:1 with DW) with or without AREG (depleted via immunoprecipitation) or with exogenous AREG at a concentration of 90 pg/mL for 7h and 48h. Fluorescence intensity was detected at the Nikon Eclipse Ti in time-lapse and quantified using the plugin RGB measure on ImageJ software (ImageJ 1.53k, NIH, Bethesda, MD, USA).

#### Immunofluorescence

Immunofluorescence (IF) was adopted to verify YAP intracellular localization and cytoskeletal modifications in AM and AMs samples. More in detail, tissues were fixed with 4% paraformaldehyde for 1h. Then, the membranes were permeabilized with PBS/Triton X-100 0.1% (#T8787; Sigma-Aldrich, St. Louis, MO, USA) for 10 minutes at room temperature (RT). Following a non-specific blocking with PBS/BSA 1% (#P3813; Sigma-Aldrich, St. Louis, MO, USA) -Tween 20 0.05% (#600481; CARLO ERBA Reagents S.r.l., Milan, Italy) for 1h at RT, YAP primary antibody (diluted 1:200 in PBS/BSA 1%-Tween 20 0.05%, #SAB2108066; Sigma-Aldrich, St. Louis, MO, USA) was incubated overnight at 4°C. Anti-rabbit Alexa Fluor 488 (#AB150077; Molecular Probes, Göteborg, Sweden) secondary antibody, diluted 1:200 in PBS/BSA 1%-Tween 20 0.05% for 1h at RT was utilized to retrieve the antigen. Afterward, the samples were incubated with Phalloidin TRITC antibody (diluted 1:10 in PBS, (#P1941; Sigma-Aldrich, St. Louis, MO, USA) for 20 minutes. The staining of the nuclei was performed with DAPI (diluted 1:2000 in PBS; #D9542; Sigma-Aldrich, St. Louis, MO, USA) for 10 minutes at RT. At each step, the membranes were maintained in gentle shaking to guarantee the homogeneous distribution of the solutions. To obtain negative controls of the reactions, primary antibodies were omitted. The image acquisition was performed under a Nikon Ar1 laser confocal scanning microscope (Nikon, Düsseldorf, Germany) equipped with the NIS-Element software 4.40 (Nikon, Düsseldorf, Germany).

#### Nuclear/cytoplasmic extraction

In order, to quantify YAP nuclear translocation in AM under different conditions, nucleus and cytoplasm separation was achieved using the NE-PER Nuclear and Cytoplasmic Extraction Reagents (#78835; Thermo Fisher Scientific^,^ Waltham, MA, USA) following the manufacturer’s instructions.

#### Western blotting

Total protein was extracted from each sample in lysis buffer (50 mM Tris HCl pH 8, 250 mM NaCl, 5 mM EDTA, 0,1% Triton X-100 10%) with Phosphatase Inhibitor (#39055; SERVA Electrophoresis GmbH, Heidelberg, Germany) and Protease Inhibitor Cocktails (#P2714; Sigma-Aldrich, St. Louis, MO, USA) diluted according to manufacturer’s instruction. Samples were put on ice for 30 minutes and then centrifuged at 12,000 x g for 12 minutes at 4°C. The supernatant was collected, and 5 μL was used to determine protein concentration with Quick Start™ Bradford 1x Dye Reagent (#5000205; Bio-Rad Laboratories, Milan, Italy). Afterward, a total of 30 μg of protein was separated by running a precast gel with a density gradient of 4-15% (#4568083; mini-PROTEAN precast gel, Bio-Rad Laboratories, Milan, Italy), and then transferred to nitrocellulose membranes (#1620145; Bio-Rad Laboratories, Milan, Italy) by using Trans-Blot Turbo 5x Transfer Buffer (#10026938; Bio-Rad Laboratories, Milan, Italy) and Trans-Blot Turbo Transfer (Bio-Rad Laboratories, Milan, Italy). Membranes were subsequently incubated with Every blot blocking solution (#12010020; Bio-Rad Laboratories, Milan, Italy) for 5 min. Primary antibodies for p-SMAD2 (1:1000, #3108; Cell Signaling Technology, Danvers, MA, USA), SMAD2/3 (1:1000, #3102; Cell Signaling Technology, Danvers, MA, USA), p-FAK (1:500, #bs-3164R; Bioss Inc., Woburn, MA, USA), FAK (1:200, # AF4467; R&D Systems, Inc., MN, USA), β-Actin (1:1000, #SC7210; Santa Cruz Biotechnology, Inc., Dallas, TX, USA), COX2 (1:300, #AB23672; Abcam Limited, Cambridge, UK), PGE_2_S (1:500, #160145; Cayman Chemical Company, Ann Arbor, MI, USA), AREG (1:200, #AF262; R&D Systems, Inc., MN, USA), YAP (1:1000, #14074; Cell Signaling Technology, Danvers, MA, USA), p-YAP (1:250, #PA5-121279; Invitrogen, Thermo Fisher Scientific, Waltham, MA, USA), a-YAP (1:1000, #29495; Cell Signaling Technology, Danvers, MA, USA), LATS (1:1.000, #3477; Cell Signaling Technology, Danvers, MA, USA), p-LATS (1:1.000, #9157; Cell Signaling Technology, Danvers, MA, USA) were diluted in 1X TBS 1% Casein Blocker (#1610782; Bio-Rad Laboratories, Milan, Italy) and incubated overnight at 4°C. Specific secondary HRP antibodies (1:10.000; Cell Signaling Technology, Danvers, MA, USA) were diluted in the solution mentioned above and incubated for 1h at RT. ClarityMax ECL reagent (#1705062; Bio-Rad Laboratories, Milan, Italy) was used to visualize the target protein and ChemiDoc MP Imaging System detected the chemiluminescent signal (Bio-Rad Laboratories, Milan, Italy). Densitometric analysis was performed using ImageJ (ImageJ 1.53k, NIH, Bethesda, MD, USA). Data were normalized to tubulin (1:2.000, #3873; Cell Signaling Technology, Danvers, MA, USA), used as a cytoplasmic housekeeping, and to H3 (1:500 #PTM-1001RM; PTM Biolabs Inc., Chicago, IL, USA) utilized as a nuclear housekeeping.

### Quantification and statistical analysis

At least three biological replicates were employed for AEC (fetus), PBMCs (animals), and zebrafish samples to verify inter-experimental variability. Each experiment was replicated three times to evaluate intra-experimental variability. The exact values of n (representing the sample size or individual animals) and the statistical significance are provided in the captions of the figures. Mean ± S.D. represented the data, with D'Agostino and Pearson tests utilized to assess normal distribution. One-way ANOVA and two tailored t-tests were employed for comparing normally distributed data, followed by Tukey post hoc analyses (GraphPad Prism 10, San Diego, CA, USA). Significance was set at a *p*-value of 0.05 or lower.

## References

[1] Medzhitov R. (2008). Origin and physiological roles of inflammation. Nature.

[2] Henderson N.C., Rieder F., Wynn T.A. (2020). Fibrosis: from mechanisms to medicines. Nature.

[3] Silini A.R., Cargnoni A., Magatti M., Pianta S., Parolini O. (2015). The Long Path of Human Placenta, and Its Derivatives, in Regenerative Medicine. Front. Bioeng. Biotechnol..

[4] Ratajczak M.Z. (2019).

[5] Zhang Q., Lai D. (2020). Application of human amniotic epithelial cells in regenerative medicine: a systematic review. Stem Cell Res. Ther..

[6] Miki T. (2018). Stem cell characteristics and the therapeutic potential of amniotic epithelial cells. Am. J. Reprod. Immunol..

[7] Nygren J.M., Jovinge S., Breitbach M., Säwén P., Röll W., Hescheler J., Taneera J., Fleischmann B.K., Jacobsen S.E.W. (2004). Bone marrow-derived hematopoietic cells generate cardiomyocytes at a low frequency through cell fusion, but not transdifferentiation. Nat. Med..

[8] Terada N., Hamazaki T., Oka M., Hoki M., Mastalerz D.M., Nakano Y., Meyer E.M., Morel L., Petersen B.E., Scott E.W. (2002). Bone marrow cells adopt the phenotype of other cells by spontaneous cell fusion. Nature.

[9] Russo V., Mauro A., Peserico A., Di Giacinto O., Khatib M.E., Citeroni M.R., Rossi E., Canciello A., Mazzotti E., Barboni B. (2022). Tendon Healing Response Is Dependent on Epithelial-Mesenchymal-Tendon Transition State of Amniotic Epithelial Stem Cells. Biomedicines.

[10] Barboni B., Russo V., Gatta V., Bernabò N., Berardinelli P., Mauro A., Martelli A., Valbonetti L., Muttini A., Di Giacinto O. (2018). Therapeutic potential of hAECs for early Achilles tendon defect repair through regeneration. J. Tissue Eng. Regen. Med..

[11] Li H., Shen S., Fu H., Wang Z., Li X., Sui X., Yuan M., Liu S., Wang G., Guo Q. (2019). Immunomodulatory functions of mesenchymal stem cells in tissue engineering. Stem Cells Int..

[12] Dumont C.M., Park J., Shea L.D. (2015). Controlled release strategies for modulating immune responses to promote tissue regeneration. J. Control. Release.

[13] Silini A.R., Magatti M., Cargnoni A., Parolini O. (2017). Is Immune Modulation the Mechanism Underlying the Beneficial Effects of Amniotic Cells and Their Derivatives in Regenerative Medicine?. Cell Transplant..

[14] Toda A., Okabe M., Yoshida T., Nikaido T. (2007). The Potential of Amniotic Membrane/Amnion-Derived Cells for Regeneration of Various Tissues. J. Pharmacol. Sci..

[15] Fathi I., Miki T. (2021). Human Amniotic Epithelial Cells Secretome: Components, Bioactivity, and Challenges. Front. Med..

[16] Cerverò-Varona A., Canciello A., Peserico A., Haidar Montes A.A., Citeroni M.R., Mauro A., Russo V., Moffa S., Pilato S., Di Giacomo S. (2023). Graphene oxide accelerates TGFβ-mediated epithelial-mesenchymal transition and stimulates pro-inflammatory immune response in amniotic epithelial cells. Mater. Today. Bio.

[17] Di Lollo V., Canciello A., Peserico A., Orsini M., Russo V., Cerveró-Varona A., Dufrusine B., El Khatib M., Curini V., Mauro A. (2023). Unveiling the immunomodulatory shift: Epithelial-mesenchymal transition Alters immune mechanisms of amniotic epithelial cells. iScience.

[18] Rossi D., Pianta S., Magatti M., Sedlmayr P., Parolini O. (2012). Characterization of the Conditioned Medium from Amniotic Membrane Cells: Prostaglandins as Key Effectors of Its Immunomodulatory Activity. PLoS One.

[19] Li H., Niederkorn J.Y., Neelam S., Mayhew E., Word R.A., McCulley J.P., Alizadeh H. (2005). Immunosuppressive Factors Secreted by Human Amniotic Epithelial Cells. Invest. Ophthalmol. Vis. Sci..

[20] Magatti M., Caruso M., De Munari S., Vertua E., De D., Manuelpillai U., Parolini O. (2015). Human Amniotic Membrane-Derived Mesenchymal and Epithelial Cells Exert Different Effects on Monocyte-Derived Dendritic Cell Differentiation and Function. Cell Transplant..

[21] Magatti M., De Munari S., Vertua E., Nassauto C., Albertini A., Wengler G.S., Parolini O. (2009). Amniotic mesenchymal tissue cells inhibit dendritic cell differentiation of peripheral blood and amnion resident monocytes. Cell Transplant..

[22] Banas R., Miller C., Guzik L., Zeevi A. (2014). Amnion-derived multipotent progenitor cells inhibit blood monocyte differentiation into mature dendritic cells. Cell Transplant..

[23] Magatti M., De Munari S., Vertua E., Gibelli L., Wengler G.S., Parolini O. (2008). Human Amnion Mesenchyme Harbors Cells with Allogeneic T-Cell Suppression and Stimulation Capabilities. Stem Cell..

[24] Barboni B., Russo V., Curini V., Martelli A., Berardinelli P., Mauro A., Mattioli M., Marchisio M., Bonassi Signoroni P., Parolini O., Colosimo A. (2014). Gestational stage affects amniotic epithelial cells phenotype, methylation status, immunomodulatory and stemness properties. Stem Cell Rev. Rep..

[25] Mauro A., Russo V., Di Marcantonio L., Berardinelli P., Martelli A., Muttini A., Mattioli M., Barboni B. (2016). M1 and M2 macrophage recruitment during tendon regeneration induced by amniotic epithelial cell allotransplantation in ovine. Res. Vet. Sci..

[26] Yen J.-H., Kocieda V.P., Jing H., Ganea D. (2011). Prostaglandin E2 Induces Matrix Metalloproteinase 9 Expression in Dendritic Cells through Two Independent Signaling Pathways Leading to Activator Protein 1 (AP-1) Activation. J. Biol. Chem..

[27] Bao Y.-S., Zhang P., Xie R.-J., Wang M., Wang Z.-Y., Zhou Z., Zhai W.-J., Feng S.-Z., Han M.-Z. (2011). The regulation of CD4+ T cell immune responses toward Th2 cell development by prostaglandin E2. Int. Immunopharmacol..

[28] Berasain C., García-Trevijano E.R., Castillo J., Erroba E., Lee D.C., Prieto J., Avila M.A. (2005). Amphiregulin: An early trigger of liver regeneration in mice. Gastroenterology.

[29] Ko J.H., Kim H.J., Jeong H.J., Lee H.J., Oh J.Y. (2020). Mesenchymal Stem and Stromal Cells Harness Macrophage-Derived Amphiregulin to Maintain Tissue Homeostasis. Cell Rep..

[30] Fang L., Sun Y.-P., Cheng J.-C. (2023). The role of amphiregulin in ovarian function and disease. Cell. Mol. Life Sci..

[31] Yu Y., Fang L., Wang S., Li Y., Guo Y., Sun Y.-P. (2019). Amphiregulin promotes trophoblast invasion and increases MMP9/TIMP1 ratio through ERK1/2 and Akt signal pathways. Life Sci..

[32] Cadenas J., Poulsen L.C., Nikiforov D., Grøndahl M.L., Kumar A., Bahnu K., Englund A.L.M., Malm J., Marko-Varga G., Pla I. (2023). Regulation of human oocyte maturation *in vivo* during the final maturation of follicles. Hum. Reprod..

[33] Cheng J.-C., Meng Q., Zhang Q., Zhang L., Chen J., Song T., Fang L., Sun Y.-P. (2023). WNK1 mediates amphiregulin-induced MMP9 expression and cell invasion in human extravillous trophoblast cells. Mol. Cell. Endocrinol..

[34] Monticelli L.A., Sonnenberg G.F., Abt M.C., Alenghat T., Ziegler C.G.K., Doering T.A., Angelosanto J.M., Laidlaw B.J., Yang C.Y., Sathaliyawala T. (2011). Innate lymphoid cells promote lung-tissue homeostasis after infection with influenza virus. Nat. Immunol..

[35] Zaiss D.M.W., Gause W.C., Osborne L.C., Artis D. (2015). Emerging Functions of Amphiregulin in Orchestrating Immunity, Inflammation, and Tissue Repair. Immunity.

[36] Burzyn D., Kuswanto W., Kolodin D., Shadrach J.L., Cerletti M., Jang Y., Sefik E., Tan T.G., Wagers A.J., Benoist C., Mathis D. (2013). A special population of regulatory T cells potentiates muscle repair. Cell.

[37] Jamieson A.M., Pasman L., Yu S., Gamradt P., Homer R.J., Decker T., Medzhitov R. (2013). Role of tissue protection in lethal respiratory viral-bacterial coinfection. Science.

[38] Zhang L., Zhang W., Li Z., Lin S., Zheng T., Hao B., Hou Y., Zhang Y., Wang K., Qin C. (2022). Mitochondria dysfunction in CD8+ T cells as an important contributing factor for cancer development and a potential target for cancer treatment: a review. J. Exp. Clin. Cancer Res..

[39] Vaeth M., Feske S. (2018). NFAT control of immune function: New Frontiers for an Abiding Trooper. F1000Res..

[40] Yoshida H., Nishina H., Takimoto H., Marengère L.E.M., Wakeham A.C., Bouchard D., Kong Y.-Y., Ohteki T., Shahinian A., Bachmann M. (1998). The Transcription Factor NF-ATc1 Regulates Lymphocyte Proliferation and Th2 Cytokine Production. Immunity.

[41] Mognol G.P., Carneiro F.R.G., Robbs B.K., Faget D.V., Viola J.P.B. (2016). Cell cycle and apoptosis regulation by NFAT transcription factors: new roles for an old player. Cell Death Dis..

[42] Jutz S., Leitner J., Schmetterer K., Doel-Perez I., Majdic O., Grabmeier-Pfistershammer K., Paster W., Huppa J.B., Steinberger P. (2016). Assessment of costimulation and coinhibition in a triple parameter T cell reporter line: Simultaneous measurement of NF-κB, NFAT and AP-1. J. Immunol. Methods.

[43] Lee J.-U., Kim L.-K., Choi J.-M. (2018). Revisiting the Concept of Targeting NFAT to Control T Cell Immunity and Autoimmune Diseases. Front. Immunol..

[44] Macian F. (2005). NFAT proteins: key regulators of T-cell development and function. Nat. Rev. Immunol..

[45] Beer L., Zimmermann M., Mitterbauer A., Ellinger A., Gruber F., Narzt M.-S., Zellner M., Gyöngyösi M., Madlener S., Simader E. (2015). Analysis of the Secretome of Apoptotic Peripheral Blood Mononuclear Cells: Impact of Released Proteins and Exosomes for Tissue Regeneration. Sci. Rep..

[46] Maraldi T., Beretti F., Guida M., Zavatti M., De Pol A. (2015). Role of Hepatocyte Growth Factor in the Immunomodulation Potential of Amniotic Fluid Stem Cells. Stem Cells Transl. Med..

[47] Wang L., Zhao Y., Shi S. (2012). Interplay between Mesenchymal Stem Cells and Lymphocytes: Implications for Immunotherapy and Tissue Regeneration. J. Dent. Res..

[48] Kulesza A., Paczek L., Burdzinska A. (2023). The Role of COX-2 and PGE2 in the Regulation of Immunomodulation and Other Functions of Mesenchymal Stromal Cells. Biomedicines.

[49] Finetti F., Paradisi L., Bernardi C., Pannini M., Trabalzini L. (2023). Cooperation between Prostaglandin E2 and Epidermal Growth Factor Receptor in Cancer Progression: A Dual Target for Cancer Therapy. Cancers.

[50] Donnini S., Finetti F., Solito R., Terzuoli E., Sacchetti A., Morbidelli L., Patrignani P., Ziche M. (2007). EP2 prostanoid receptor promotes squamous cell carcinoma growth through epidermal growth factor receptor transactivation and iNOS and ERK1/2 pathways. FASEB J..

[51] Sales K.J., Maudsley S., Jabbour H.N. (2004). Elevated Prostaglandin EP2 Receptor in Endometrial Adenocarcinoma Cells Promotes Vascular Endothelial Growth Factor Expression via Cyclic 3′,5′-Adenosine Monophosphate-Mediated Transactivation of the Epidermal Growth Factor Receptor and Extracellular Signal-Regulated Kinase 1/2 Signaling Pathways. Mol. Endocrinol..

[52] Ding Y.-B., Shi R.-H., Tong J.-D., Li X.-Y., Zhang G.-X., Xiao W.-M., Yang J.-G., Bao Y., Wu J., Yan Z.-G., Wang X.H. (2005). PGE2 up-regulates vascular endothelial growth factor expression in MKN28 gastric cancer cells via epidermal growth factor receptor signaling system. Exp. Oncol..

[53] Fernández-Martínez A.B., Lucio-Cazaña J. (2015). Intracellular EP2 prostanoid receptor promotes cancer-related phenotypes in PC3 cells. Cell. Mol. Life Sci..

[54] Oshima H., Popivanova B.K., Oguma K., Kong D., Ishikawa T., Oshima M. (2011). Activation of epidermal growth factor receptor signaling by the prostaglandin E _2_ receptor EP4 pathway during gastric tumorigenesis. Cancer Sci..

[55] Bazzani L., Donnini S., Finetti F., Christofori G., Ziche M. (2017). PGE2/EP3/SRC signaling induces EGFR nuclear translocation and growth through EGFR ligands release in lung adenocarcinoma cells. Oncotarget.

[56] Tveteraas I.H., Müller K.M., Aasrum M., Ødegård J., Dajani O., Guren T., Sandnes D., Christoffersen T. (2012). Mechanisms involved in PGE2-induced transactivation of the epidermal growth factor receptor in MH1C1 hepatocarcinoma cells. J. Exp. Clin. Cancer Res..

[57] Minutti C.M., Modak R.V., Macdonald F., Li F., Smyth D.J., Dorward D.A., Blair N., Husovsky C., Muir A., Giampazolias E. (2019). A Macrophage-Pericyte Axis Directs Tissue Restoration via Amphiregulin-Induced Transforming Growth Factor Beta Activation. Immunity.

[58] Lian I., Kim J., Okazawa H., Zhao J., Zhao B., Yu J., Chinnaiyan A., Israel M.A., Goldstein L.S.B., Abujarour R. (2010). The role of YAP transcription coactivator in regulating stem cell self-renewal and differentiation. Genes Dev..

[59] Yoshii H., Kajiya M., Yoshino M., Morimoto S., Horikoshi S., Tari M., Motoike S., Iwata T., Ouhara K., Ando T. (2024). Mechanosignaling YAP/TAZ-TEAD Axis Regulates the Immunomodulatory Properties of Mesenchymal Stem Cells. Stem Cell Rev. Rep..

[60] Wan S., Fu X., Ji Y., Li M., Shi X., Wang Y. (2018). FAK- and YAP/TAZ dependent mechanotransduction pathways are required for enhanced immunomodulatory properties of adipose-derived mesenchymal stem cells induced by aligned fibrous scaffolds. Biomaterials.

[61] Basu A., Paul M.K., Alioscha-Perez M., Grosberg A., Sahli H., Dubinett S.M., Weiss S. (2022). Statistical parametrization of cell cytoskeleton reveals lung cancer cytoskeletal phenotype with partial EMT signature. Commun. Biol..

[62] Sebag S.C., Bastarache J.A., Ware L.B. (2013). Mechanical Stretch Inhibits Lipopolysaccharide-induced Keratinocyte-derived Chemokine and Tissue Factor Expression While Increasing Procoagulant Activity in Murine Lung Epithelial Cells. J. Biol. Chem..

[63] Massou S., Nunes Vicente F., Wetzel F., Mehidi A., Strehle D., Leduc C., Voituriez R., Rossier O., Nassoy P., Giannone G. (2020). Cell stretching is amplified by active actin remodelling to deform and recruit proteins in mechanosensitive structures. Nat. Cell Biol..

[64] Gibault F., Bailly F., Corvaisier M., Coevoet M., Huet G., Melnyk P., Cotelle P. (2017). Molecular Features of the YAP Inhibitor Verteporfin: Synthesis of Hexasubstituted Dipyrrins as Potential Inhibitors of YAP/TAZ, the Downstream Effectors of the Hippo Pathway. ChemMedChem.

[65] Santucci M., Vignudelli T., Ferrari S., Mor M., Scalvini L., Bolognesi M.L., Uliassi E., Costi M.P. (2015). The Hippo Pathway and YAP/TAZ–TEAD Protein–Protein Interaction as Targets for Regenerative Medicine and Cancer Treatment: Miniperspective. J. Med. Chem..

[66] Zhao B., Li L., Tumaneng K., Wang C.-Y., Guan K.-L. (2010). A coordinated phosphorylation by Lats and CK1 regulates YAP stability through SCF ^β-TRCP^. Genes Dev..

[67] Kastan N., Gnedeva K., Alisch T., Petelski A.A., Huggins D.J., Chiaravalli J., Aharanov A., Shakked A., Tzahor E., Nagiel A. (2021). Small-molecule inhibition of Lats kinases may promote Yap-dependent proliferation in postmitotic mammalian tissues. Nat. Commun..

[68] Kastan N.R., Oak S., Liang R., Baxt L., Myers R.W., Ginn J., Liverton N., Huggins D.J., Pichardo J., Paul M. (2022). Development of an improved inhibitor of Lats kinases to promote regeneration of mammalian organs. Proc. Natl. Acad. Sci. USA.

[69] Bae S.J., Luo X. (2018). Activation mechanisms of the Hippo kinase signaling cascade. Biosci. Rep..

[70] Chen L., Loh P.G., Song H. (2010). Structural and functional insights into the TEAD-YAP complex in the Hippo signaling pathway. Protein Cell.

[71] Zhao B., Ye X., Yu J., Li L., Li W., Li S., Yu J., Lin J.D., Wang C.-Y., Chinnaiyan A.M. (2008). TEAD mediates YAP-dependent gene induction and growth control. Genes Dev..

[72] Dupont S., Morsut L., Aragona M., Enzo E., Giulitti S., Cordenonsi M., Zanconato F., Le Digabel J., Forcato M., Bicciato S. (2011). Role of YAP/TAZ in mechanotransduction. Nature.

[73] Angé M., Castanares-Zapatero D., De Poortere J., Dufeys C., Courtoy G.E., Bouzin C., Quarck R., Bertrand L., Beauloye C., Horman S. (2020). α1AMP-Activated Protein Kinase Protects against Lipopolysaccharide-Induced Endothelial Barrier Disruption via Junctional Reinforcement and Activation of the p38 MAPK/HSP27 Pathway. Int. J. Mol. Sci..

[74] Li T., Wen Y., Lu Q., Hua S., Hou Y., Du X., Zheng Y., Sun S. (2023). MST1/2 in inflammation and immunity. Cell Adh. Migr..

[75] Wang S., Zhou L., Ling L., Meng X., Chu F., Zhang S., Zhou F. (2020). The Crosstalk Between Hippo-YAP Pathway and Innate Immunity. Front. Immunol..

[76] Ueta M., Kweon M.-N., Sano Y., Sotozono C., Yamada J., Koizumi N., Kiyono H., Kinoshita S. (2002). Immunosuppressive properties of human amniotic membrane for mixed lymphocyte reaction. Clin. Exp. Immunol..

[77] Bailo M., Soncini M., Vertua E., Signoroni P.B., Sanzone S., Lombardi G., Arienti D., Calamani F., Zatti D., Paul P. (2004). Engraftment Potential of Human Amnion and Chorion Cells Derived from Term Placenta. Transplantation.

[78] Pratama G., Vaghjiani V., Tee J.Y., Liu Y.H., Chan J., Tan C., Murthi P., Gargett C., Manuelpillai U. (2011). Changes in Culture Expanded Human Amniotic Epithelial Cells: Implications for Potential Therapeutic Applications. PLoS One.

[79] Kang N.-H., Hwang K.-A., Kim S.U., Kim Y.-B., Hyun S.-H., Jeung E.-B., Choi K.-C. (2012). Potential antitumor therapeutic strategies of human amniotic membrane and amniotic fluid-derived stem cells. Cancer Gene Ther..

[80] Parolini O., Souza-Moreira L., O’Valle F., Magatti M., Hernandez-Cortes P., Gonzalez-Rey E., Delgado M. (2014). Therapeutic Effect of Human Amniotic Membrane–Derived Cells on Experimental Arthritis and Other Inflammatory Disorders. Arthritis Rheumatol..

[81] Vasandan A.B., Jahnavi S., Shashank C., Prasad P., Kumar A., Prasanna S.J. (2016). Human Mesenchymal stem cells program macrophage plasticity by altering their metabolic status via a PGE_2_-dependent mechanism. Sci. Rep..

[82] Harris S.G., Padilla J., Koumas L., Ray D., Phipps R.P. (2002). Prostaglandins as modulators of immunity. Trends Immunol..

[83] Scher J.U., Pillinger M.H. (2009). The Anti-Inflammatory Effects of Prostaglandins. J. Investig. Med..

[84] Kvirkvelia N., Vojnovic I., Warner T.D., Athie-Morales V., Free P., Rayment N., Chain B.M., Rademacher T.W., Lund T., Roitt I.M., Delves P.J. (2002). Placentally derived prostaglandin E2 acts via the EP4 receptor to inhibit IL-2-dependent proliferation of CTLL-2 T cells. Clin. Exp. Immunol..

[85] Buchanan F.G., Wang D., Bargiacchi F., DuBois R.N. (2003). Prostaglandin E2 Regulates Cell Migration via the Intracellular Activation of the Epidermal Growth Factor Receptor. J. Biol. Chem..

[86] Güler Ö., Özer A., Seyithanoğlu M., Yaman F.N., Şahpaz Kurşun H.N. (2021). Serum amphiregulin and cerebellin-1 levels in severe preeclampsia. J. Matern. Fetal Neonatal Med..

[87] Lee D.-S., Yanagimoto Ueta Y., Suzuki H. (2006). Expression of Amphiregulin During the Pre- and Post-implantation Period in the Mouse Reproductive Tract. J. Reprod. Dev..

[88] Kang Y.-J., Mbonye U.R., DeLong C.J., Wada M., Smith W.L. (2007). Regulation of intracellular cyclooxygenase levels by gene transcription and protein degradation. Prog. Lipid Res..

[89] Streicher K.L., Willmarth N.E., Garcia J., Boerner J.L., Dewey T.G., Ethier S.P. (2007). Activation of a nuclear factor kappaB/interleukin-1 positive feedback loop by amphiregulin in human breast cancer cells. Mol. Cancer Res..

[90] Chen Y.-T., Hou C.-H., Hou S.-M., Liu J.-F. (2014). The Effects of Amphiregulin Induced MMP-13 Production in Human Osteoarthritis Synovial Fibroblast. Mediators Inflamm..

[91] Heo Y.J., Lee N., Choi S.-E., Jeon J.Y., Han S.J., Kim D.J., Kang Y., Lee K.W., Kim H.J. (2023). Amphiregulin Induces iNOS and COX-_2_ Expression through NF-κB and MAPK Signaling in Hepatic Inflammation. Mediators Inflamm..

[92] Rausch V., Hansen C.G. (2020). The Hippo Pathway, YAP/TAZ, and the Plasma Membrane. Trends Cell Biol..

[93] Dasgupta I., McCollum D. (2019). Control of cellular responses to mechanical cues through YAP/TAZ regulation. J. Biol. Chem..

[94] Kim H.-B., Kim M., Park Y.-S., Park I., Kim T., Yang S.-Y., Cho C.J., Hwang D., Jung J.-H., Markowitz S.D. (2017). Prostaglandin E_2_ Activates YAP and a Positive-Signaling Loop to Promote Colon Regeneration After Colitis but Also Carcinogenesis in Mice. Gastroenterology.

[95] Lee N.-H., Kim S.J., Hyun J. (2021). MicroRNAs Regulating Hippo-YAP Signaling in Liver Cancer. Biomedicines.

[96] Yao L., He J., Li B., Yan M., Wang H., Tan L., Liu M., Lv X., Lv H., Zhang X. (2019). Regulation of YAP by Mammalian Target of Rapamycin Complex 1 in Endothelial Cells Controls Blood Pressure Through COX-_2_/mPGES-1/PGE_2_ Cascade. Hypertension.

[97] Bosveld F., Markova O., Guirao B., Martin C., Wang Z., Pierre A., Balakireva M., Gaugue I., Ainslie A., Christophorou N. (2016). Epithelial tricellular junctions act as interphase cell shape sensors to orient mitosis. Nature.

[98] Charras G., Yap A.S. (2018). Tensile Forces and Mechanotransduction at Cell–Cell Junctions. Curr. Biol..

[99] Malinova T.S., Huveneers S. (2018). Sensing of Cytoskeletal Forces by Asymmetric Adherens Junctions. Trends Cell Biol..

[100] Yap A.S., Duszyc K., Viasnoff V. (2018). Mechanosensing and Mechanotransduction at Cell–Cell Junctions. Cold Spring Harb. Perspect. Biol..

[101] Kyuno D., Takasawa A., Kikuchi S., Takemasa I., Osanai M., Kojima T. (2021). Role of tight junctions in the epithelial-to-mesenchymal transition of cancer cells. Biochim. Biophys. Acta. Biomembr..

[102] Serrano-Gomez S.J., Maziveyi M., Alahari S.K. (2016). Regulation of epithelial-mesenchymal transition through epigenetic and post-translational modifications. Mol. Cancer.

[103] Mohan A.R., Sooranna S.R., Lindstrom T.M., Johnson M.R., Bennett P.R. (2007). The effect of mechanical stretch on cyclooxygenase type 2 expression and activator protein-1 and nuclear factor-kappaB activity in human amnion cells. Endocrinology.

[104] Kendal-Wright C.E. (2007). Stretching, Mechanotransduction, and Proinflammatory Cytokines in the Fetal Membranes. Reprod. Sci..

[105] Brown C.L., Meise K.S., Plowman G.D., Coffey R.J., Dempsey P.J. (1998). Cell surface ectodomain cleavage of human amphiregulin precursor is sensitive to a metalloprotease inhibitor. Release of a predominant N-glycosylated 43-kDa soluble form. J. Biol. Chem..

[106] Worthington J.J., Fenton T.M., Czajkowska B.I., Klementowicz J.E., Travis M.A. (2012). Regulation of TGFβ in the immune system: An emerging role for integrins and dendritic cells. Immunobiology.

[107] Ghamari S.-H., Abbasi-Kangevari M., Tayebi T., Bahrami S., Niknejad H. (2020). The Bottlenecks in Translating Placenta-Derived Amniotic Epithelial and Mesenchymal Stromal Cells Into the Clinic: Current Discrepancies in Marker Reports. Front. Bioeng. Biotechnol..

[108] Semenov O.V., Koestenbauer S., Riegel M., Zech N., Zimmermann R., Zisch A.H., Malek A. (2010). Multipotent mesenchymal stem cells from human placenta: critical parameters for isolation and maintenance of stemness after isolation. Am. J. Obstet. Gynecol..

[109] Mauro A., Sanyal H., Canciello A., Berardinelli P., Russo V., Bernabò N., Valbonetti L., Barboni B. (2019). In Vitro Effect of Estradiol and Progesterone on Ovine Amniotic Epithelial Cells. Stem Cells Int..

[110] Canciello A., Russo V., Berardinelli P., Bernabò N., Muttini A., Mattioli M., Barboni B. (2017). Progesterone prevents epithelial-mesenchymal transition of ovine amniotic epithelial cells and enhances their immunomodulatory properties. Sci. Rep..

[111] Benson-Martin J., Zammaretti P., Bilic G., Schweizer T., Portmann-Lanz B., Burkhardt T., Zimmermann R., Ochsenbein-Kölble N. (2006). The Young’s modulus of fetal preterm and term amniotic membranes. Eur. J. Obstet. Gynecol. Reprod. Biol..

[112] Sideris I.G., Nicolaides K.H. (1990). Amniotic Fluid Pressure during Pregnancy. Fetal Diagn. Ther..

[113] Fisk N.M., Ronderos-Dumit D., Tannirandorn Y., Nicolini U., Talbert D., Rodeck C.H. (1992). Normal amniotic pressure throughout gestation. Br. J. Obstet. Gynaecol..

[114] Flores-Espinosa P., Pineda-Torres M., Vega-Sánchez R., Estrada-Gutiérrez G., Espejel-Nuñez A., Flores-Pliego A., Maida-Claros R., Paredes-Vivas Y., Morales-Méndez I., Sosa-González I. (2014). Progesterone Elicits an Inhibitory Effect upon LPS -Induced Innate Immune Response in Pre-Labor Human Amniotic Epithelium. Am. J. Reprod. Immunol..

[115] Canciello A., Teti G., Mazzotti E., Falconi M., Russo V., Giordano A., Barboni B. (2020). Progesterone Prolongs Viability and Anti-inflammatory Functions of Explanted Preterm Ovine Amniotic Membrane. Front. Bioeng. Biotechnol..

