# Supplementary material for: Amphiregulin orchestrates the paracrine immune-suppressive function of amniotic-derived cells through its interplay with COX-2/PGE2/EP4 axis
Source: iScience. 2024 Jul 14;27(8):110508. doi: 10.1016/j.isci.2024.110508 (PMC11326934; doi:10.1016/j.isci.2024.110508)
Supplement: Document S1. Figures S1–S4 [file mmc1.pdf]

iScience, Volume *27*

Supplemental information

Amphiregulin orchestrates the paracrine immune-suppressive function of amniotic-derived cells through its interplay with COX-2/PGE_2/EP4 axis

Giuseppe Prencipe, Adrián Cerveró-Varona, Monia Perugini, Ludovica Sulcanese, Annamaria Iannetta, Arlette Alina Haidar-Montes, Johannes Stöckl, Angelo Canciello, Paolo Berardinelli, Valentina Russo, and Barbara Barboni

Supplemental Information

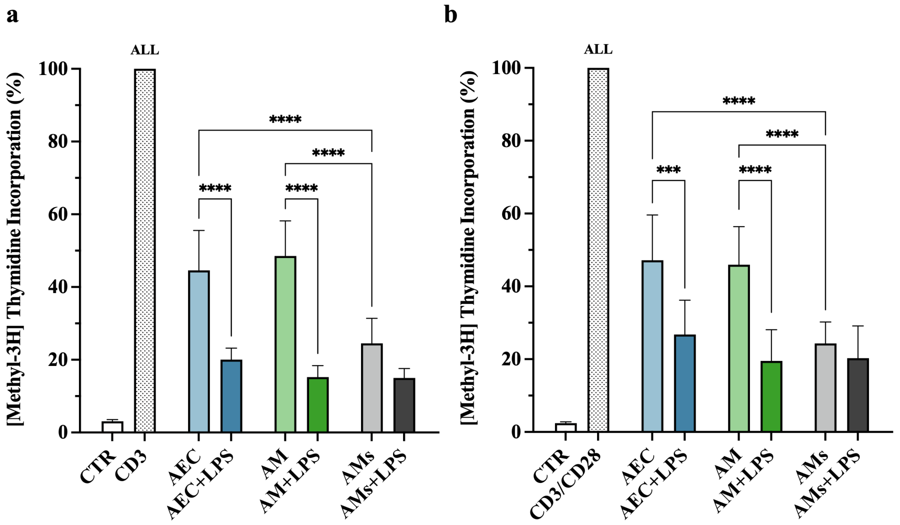

Figure S1. Amniotic-Derived Cells/Tissue' CM Effectively Suppress Human PBMCs Activation, related to Figure 1**. (**a**) CD3 and (**b**) CD3/CD28-stimulated human PBMCs treated with the different CM (± LPS) for 48h were evaluated for DNA synthesis. Data are normalized on CD3/CD28 stimulated human PBMCs (100% of [methyl-3H] thymidine incorporation). The obtained results aligned with the inhibitory profile assessed on the ovine model, thus, confirming the potential strong xeno-application of the produced CM. Data (mean ± SD) represent 3 independent sets of experiments (n = at least 3 biological replicates in each group per set; each biological replicate assayed in at least 3 technical replicates). ALL, ***, and * Statistically significant values between the different studied groups (p<0.0001, p<0.001, and p<0.0001, respectively).*

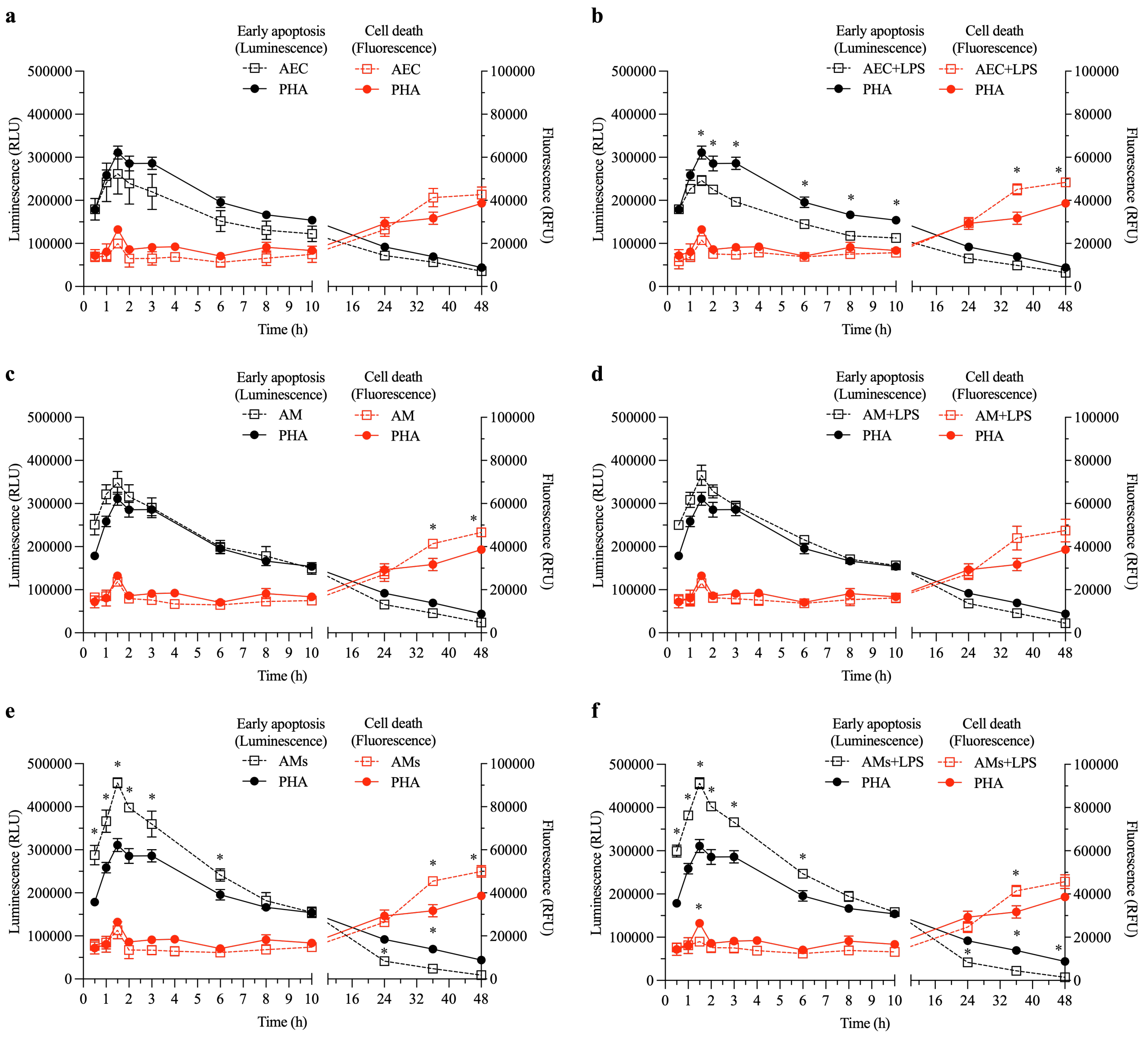

Figure S2. Amniotic-Derived Cells/Tissue' CM Effectively Induce Early Apoptosis and Cell Death on PHA-activated PBMCs, related to Figure 1*. 48h time course analysis with CM derived from (****a****) AEC, (****b****) AEC+LPS, (****c****) AM, (****d****) AM+LPS, (****e****) AMs, and (****f****) AMs+LPS.*

*Data (mean ± SD) represent 3 independent sets of experiments (n = at least 3 biological replicates in each group per set; each biological replicate assayed in at least 3 technical replicates). * Statistically significant values between the different studied groups (p<0.05).*

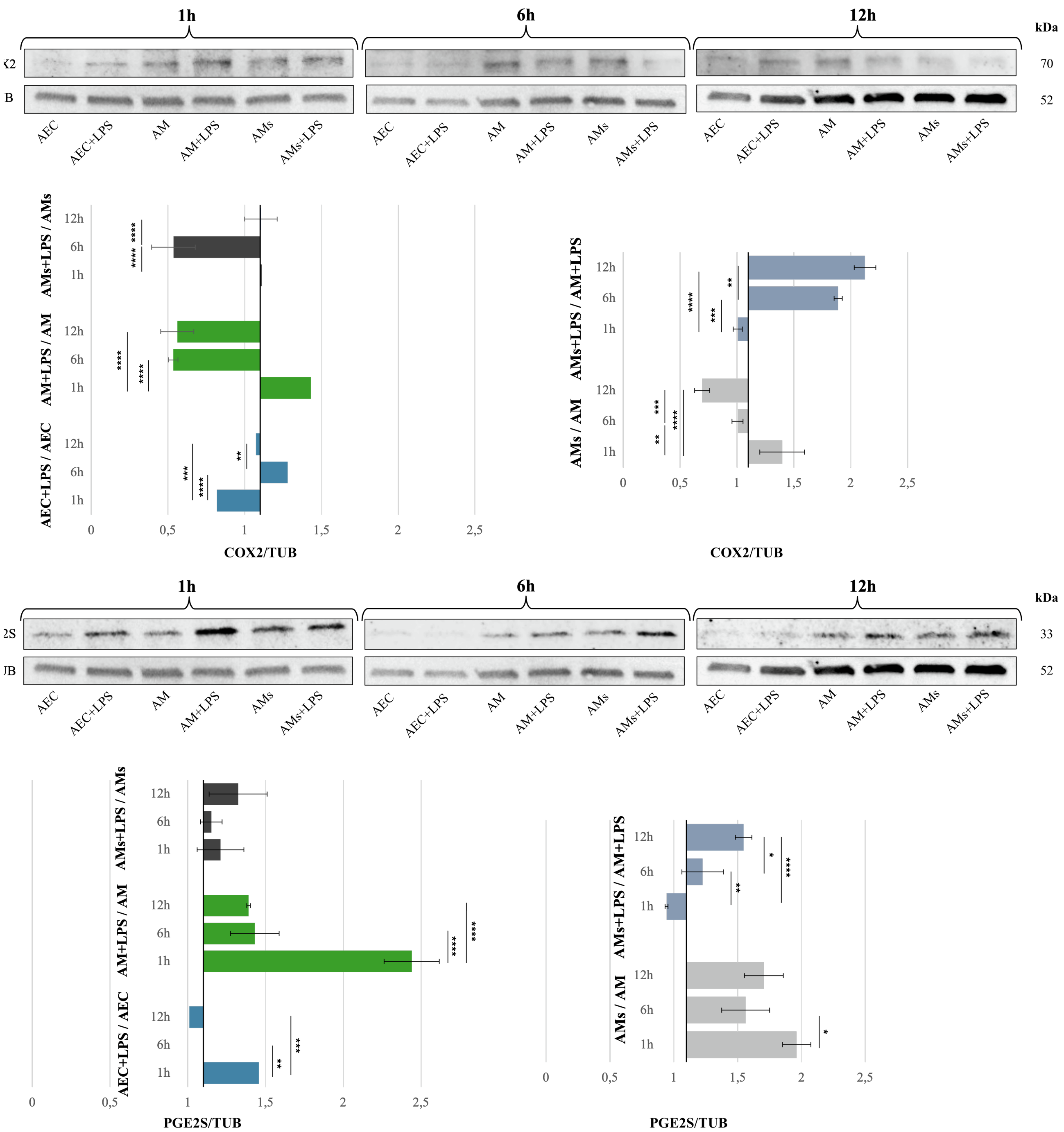

Figure S3. Amniotic-Derived Cells/Tissue (± LPS) upregulate COX2/PGE$_2$S axis, related to Figure 2. *Representative WB images and relative densitometric analysis of (**a**) COX2 and (**b**) PGE$_2$S expression relative to amniotic-derived cells/tissue (±LPS) cultured at 1, 6, and 12h. Data were normalized to the corresponding housekeeping Tubulin expression.*

*Data (mean ± SD) represent 3 independent sets of experiments (n = at least 3 biological replicates in each group per set; each biological replicate assayed in at least 3 technical replicates). *, **, ***, and **** Statistically significant values between the different studied groups (p<0.05, p<0.01, p<0.001, and p<0.0001, respectively).*

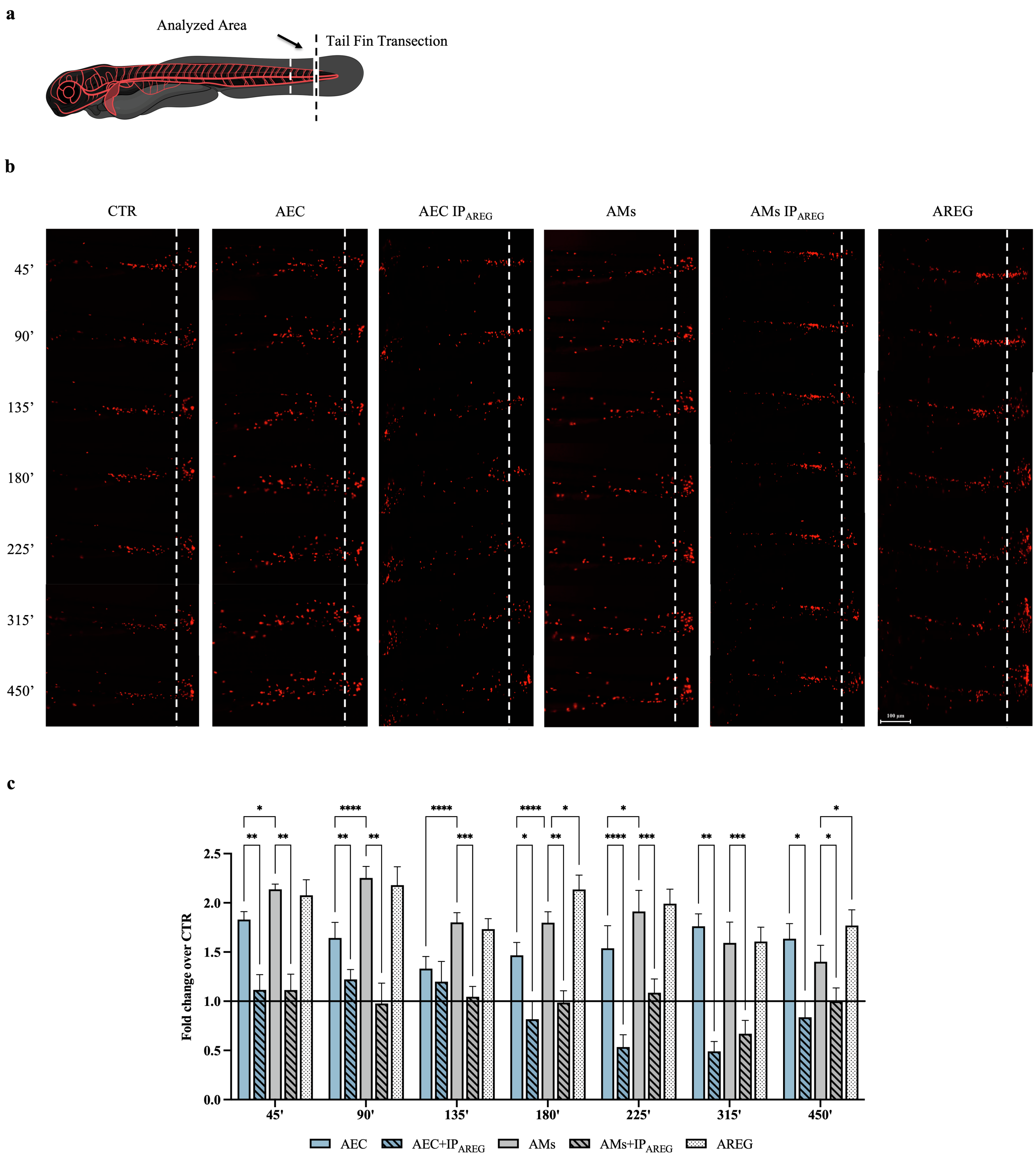

Figure S4. AREG is a Key Immune Modulator of Acute Inflammation Induced in Zebrafish Larvae, related to Figure 3*.* ***(a)*** *Illustration depicting a zebrafish larva at 72h post-fertilization (hpf).* ***(b)*** *Exemplary images of Tg(lysC:DsRed2) larvae post tail fin transection, showcasing the localization of immune cells in the tail and their accumulation at the site of injury (right of the dashed white line) in a time-lapse of 450’.* ***(c)*** *Densitometric analysis of total fluorescence intensity (TFI), presenting the percentage of immune cell proliferation detected within the wounded area at different time points.*

*Data (mean ± SD) represent 3 independent sets of experiments (n = at least 3 biological replicates in each group per set; each biological replicate assayed in at least 3 technical replicates). *, **, ***, and **** Statistically significant values between the different studied groups ($p<0.05$, $p<0.01$, $p<0.001$, and $p<0.0001$, respectively). Scale bar, 100 µm.*
